# Coronary angiography: a review of the state of the art and the evolution of angiography in cardio therapeutics

**DOI:** 10.3389/fcvm.2024.1468888

**Published:** 2024-11-25

**Authors:** Aishwarya Gurav, Pruthvi C. Revaiah, Tsung-Ying Tsai, Kotaro Miyashita, Akihiro Tobe, Asahi Oshima, Emelyne Sevestre, Scot Garg, Jean-Paul Aben, Johan H. C. Reiber, Marie Angele Morel, Cheol Whan Lee, Bon-Kwon Koo, Simone Biscaglia, Carlos Collet, Christos Bourantas, Javier Escaned, Yoshinobu Onuma, Patrick W. Serruys

**Affiliations:** ^1^ CORRIB Research Centre for Advanced Imaging and Core Laboratory, University of Galway, Galway, Ireland; ^2^ Department of Cardiology, Royal Blackburn Hospital, Blackburn, United Kingdom; ^3^ Pie Medical Imaging BV, Maastricht, Netherlands; ^4^ Department of Radiology, Leiden University Medical Center, Leiden, Netherlands; ^5^ Medis Medical Imaging Systems BV, Leiden, Netherlands; ^6^ Division of Cardiology, Department of Internal Medicine, Asan Medical Center, University of Ulsan College of Medicine, Seoul, Republic of Korea; ^7^ Department of Internal Medicine and Cardiovascular Center, Seoul National University Hospital, Seoul, Republic of Korea; ^8^ Cardiology Unit, Azienda Ospedaliero Universitaria di Ferrara, Ferrara, Italy; ^9^ Cardiovascular Center Aalst, OLV Clinic, Aalst, Belgium; ^10^ Department of Cardiology, Barts Heart Center, Barts Health NHS Trust, London, United Kingdom; ^11^ Cardiovascular Devices Hub, Centre for Cardiovascular Medicine and Devices, William Harvey Research Institute, Queen Mary University of London, London, United Kingdom; ^12^ Hospital Clínico San Carlos IDISSC, Complutense University of Madrid and CIBER-CV, Madrid, Spain

**Keywords:** coronary angiography, angiography-based fractional flow reserve, computed tomography coronary angiography, percutaneous coronary intervention, pullback pressure gradient, wall shear stress (WSS)

## Abstract

Traditionally, coronary angiography was restricted to visual estimation of contrast-filled lumen in coronary obstructive diseases. Over the previous decades, considerable development has been made in quantitatively analyzing coronary angiography, significantly improving its accuracy and reproducibility.  Notably, the integration of artificial intelligence (AI) and machine learning into quantitative coronary angiography (QCA) holds promise for further enhancing diagnostic accuracy and predictive capabilities. In addition, non-invasive fractional flow reserve (FFR) indices, including computed tomography-FFR, have emerged as valuable tools, offering precise physiological assessment of coronary artery disease without the need for invasive procedures. These innovations allow for a more comprehensive evaluation of disease severity and aid in guiding revascularization decisions. This review traces the development of QCA technologies over the years, highlighting key milestones and current advancements. It also explores prospects that could revolutionize the field, such as AI integration and improved imaging techniques. By addressing both historical context and future directions, the article underscores the ongoing evolution of QCA and its critical role in the accurate assessment and management of coronary artery diseases. Through continuous innovation, QCA is poised to remain at the forefront of cardiovascular diagnostics, offering clinicians invaluable tools for improving patient care.

## Introduction

Coronary angiography has continuously evolved since its inception in 1956 when Mason Sones accidentally engaged the right coronary artery of a patient (1). As a result of its huge impact on the understanding and assessment of coronary artery disease (CAD), for the first time, a diagnostic method used in cardiology could be referred to as the “gold standard.” History demonstrates that recognizing the limitations of a given modality often spurs the development of strategies to overcome these limitations, a statement that rings true in the history of coronary angiography. Visual assessment of coronary angiograms is largely subjective, exhibiting significant inter-observer and intra-observer variabilities (2). Moreover, angiography only highlights the contrast-filled lumen, or “luminogram,” while largely ignoring the total plaque burden (PB) and vessel remodeling, which are pathognomonic of obstructive coronary artery disease. In addition, angiography gives a two-dimensional (2D) representation of the lumen anatomy instead of a comprehensive three-dimensional (3D) visualization of the lumen. Therefore, accurate assessment of luminal diameter and stenosis is crucial, and consequently there has been a significant push toward developing more reproducible methods for vessel pathology assessment. In 1975, the American Heart Association established a reporting system for grading the coronary system (3). In 1978, Dr. Bruce Gregory Brown, a cardiology fellow in the lab of Dr. Hal Dodge at the University of Washington, developed a digital electronic caliper to measure coronary artery narrowing, the first quantitative angiography system, for which, following its validation in 10 stenoses from 7 patients (4), the American Heart Association awarded him the first Irvine H. Page Award.

In 1977, Andreas Grüntzig achieved another milestone that boosted coronary angiography, the introduction of balloon angioplasty (percutaneous transluminal coronary angioplasty). The need to gauge the luminal dimensions to choose the correct balloon size, and also to estimate accurately luminal loss in the process known as restenosis, paved the way for further refinements in quantitative coronary angiography (QCA). Since then, this field has been continuously evolving. This article aims to provide an overview of the development of QCA technologies over the years and explore their future prospects (see Central Illustration).

**Central Illustration:**
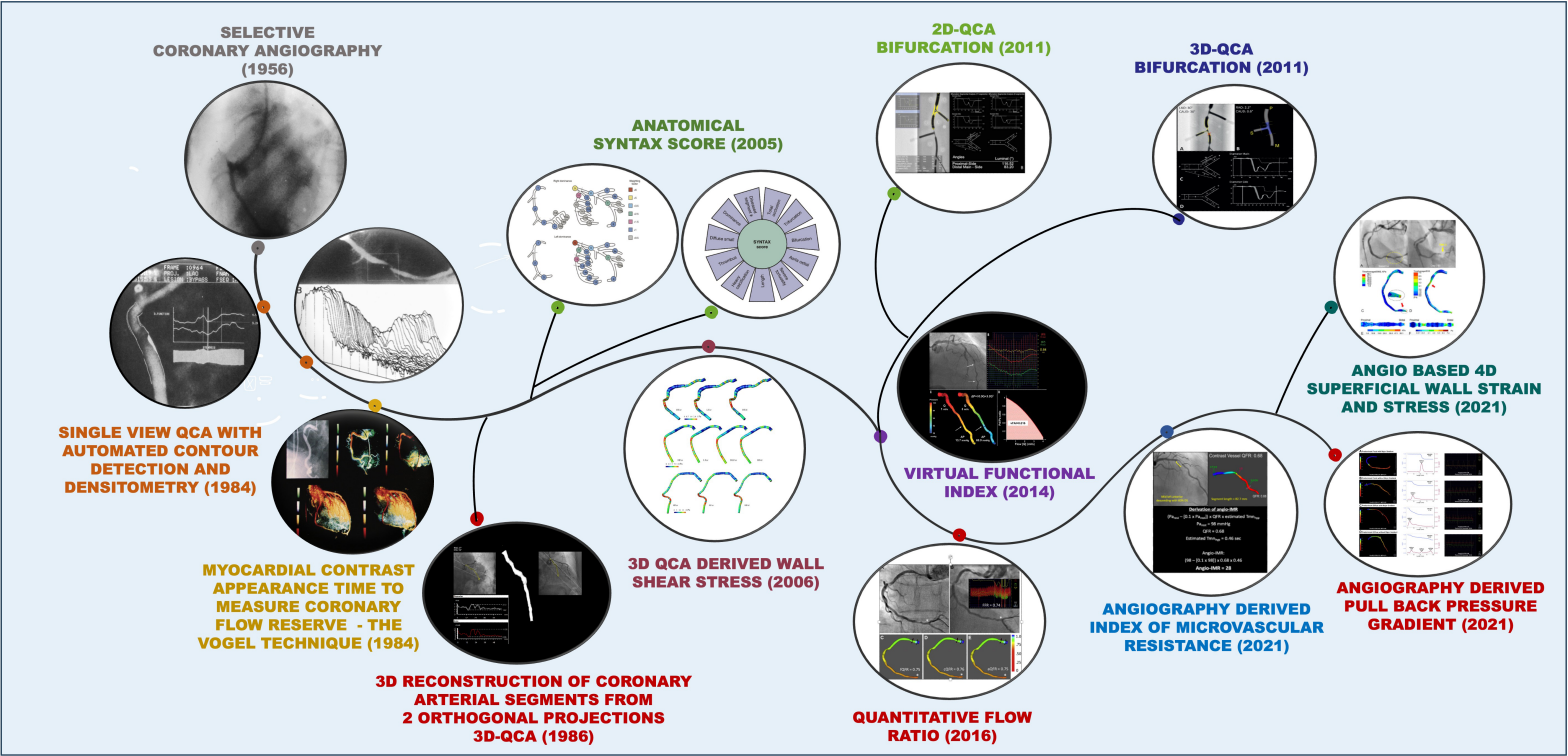
Evolution of coronary angiography over time. Coronary angiography, first introduced in 1956, has undergone significant advancements over the decades and continues to evolve. Numerous innovations have contributed to progress in this field. The illustration depicts the various technological advancements in coronary angiography, alongside their respective years of introduction.

### Conventional QCA-based on single-vessel assessment

**Figure 1:**
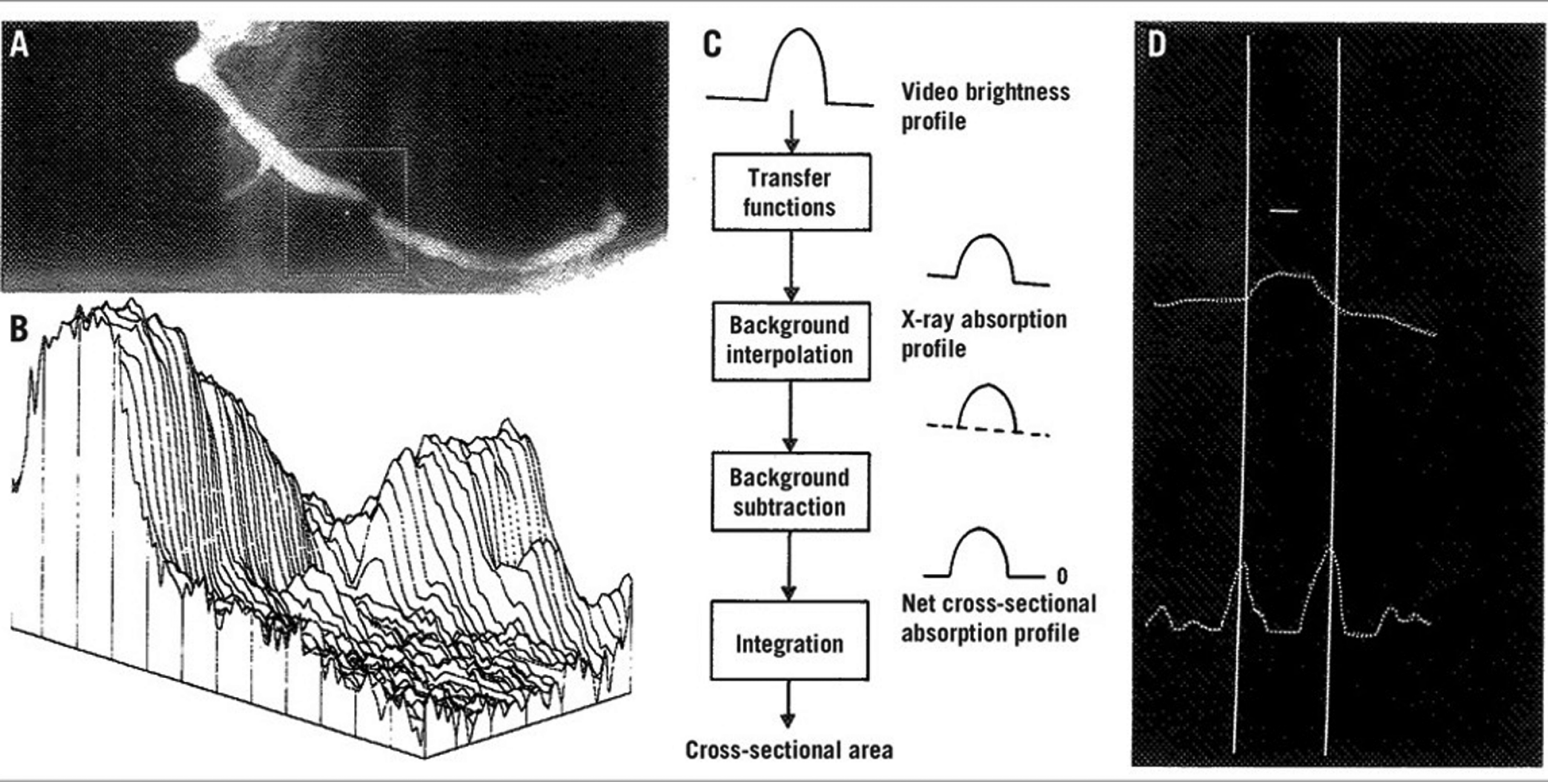
Basic aspects of the densitometric technique for quantitative coronary angiography analysis. **(A)** A matrix is placed over the area selected for analysis from the right coronary angiogram, encompassing a severe coronary obstruction. **(B)** Pseudo three-dimensional representation of the brightness information within the matrix. The coronary artery can be recognized as a mountain ridge with a deep pass at the site of the obstruction. **(C)** This flow chart of the analysis indicates the main procedures followed for the computation of the densitometric area function. **(D)** The brightness profile along one particular scanline is plotted. Positions with maximal values of the sum of the first and second derivative functions left and right of the center positions of the artery correspond with the edge positions of the artery [Reproduced from Serruys et al. (5)].

A team at the Thoraxcenter in Rotterdam, led by Professor Patrick W. Serruys and Professor Hans Reiber, developed a contour detection algorithm that continues to be a key component of modern QCA software (5–7). QCA has been crucial in evaluating new interventional techniques, especially in assessing the effectiveness of innovative percutaneous coronary interventions (PCI) such as balloon angioplasty, directional coronary atherectomy, rotational atherectomy, bare metal stents (BMS), excimer laser, drug-eluting stents (DES), and drug-coated balloons (8–11). QCA has introduced numerous surrogate endpoints, such as acute gain, late lumen loss (LLL), and percentage diameter stenosis (%DS), which have been correlated with clinical events in long-term follow-up studies (12). QCA of the coronary segment was performed using a computer-assisted cardiovascular angiography analysis system (CAAS) (6). A cine-video converter transformed an optically magnified section of the image, covering the segment in a chosen frame of 35 mm cinefilm, into a video format. This process involved manually defining the start and endpoint of the selected segment, after which software was developed to automatically detect the path line. Contours are then detected automatically using a weighted sum of first and second derivative functions applied to the digitalized brightness information (7). The contour detection process employs the “minimal cost contour detection” method. Lumen contours are displayed from the start to the endpoint, and similarly, the healthy, non-diseased vessel contour is reconstructed. The difference between the two contour lines provides numerous parameters, including maximum percentage stenosis at a particular site, degree of obstruction, areas of atherosclerotic plaque, and stenotic flow reserve (13, 14) (Figure 1).

When the pixel size is not in Digital Imaging and Communications in Medicine (DICOM) format, a catheter is used as a scaling device to calibrate the vessel's diameter data in absolute terms.

Calibration is performed on a non-tapering segment of the contrast-filled catheter to ensure precise vessel dimension measurements. In arteries with a focal obstructive lesion and a normal proximal or distal segment, selecting the reference region is straightforward. However, when the proximal or distal segments exhibit a mix of stenotic and ectatic areas, choosing a reference becomes challenging. To address this, an alternative method was introduced: the “interpolated percent% DS measurement,” which expresses the severity of a coronary obstruction without relying on a user-defined reference region. The key idea of this method is computer estimation of the original vessel diameter across the obstructive area, assuming no coronary disease existed. This is done using the diameter function, based on proximal and distal centerline segments. From this computed reference diameter function, the reference contours of the obstructed area can be reconstructed. The interpolated% DS is determined by comparing the minimal lumen diameter (MLD) at the obstruction with the corresponding reference value. The accuracy of this quantification method has been validated using plexiglass phantoms filled with contrast medium (15). Despite continuous vessel tapering not naturally occurring in the coronary arteries (coronary luminal diameter decreases discontinuously as a result of the emergence of multiple side branches) (16), the interpolated %DS stenosis measurement provides a pragmatic approach to conceptually merge minimal luminal and reference diameters in stenosis.

### Methods of QCA

There are two approaches proposed in the literature for QCA analysis:

1. The contour detection approach: A computer-based coronary angiography analysis system is used to perform quantitative analysis of selected coronary segments. This process requires manually defining several center positions within the segments, resulting in a smooth, continuous curve known as the centerline. Based on the centerline, the luminal borders are detected using a minimum costs algorithm.
2. Densitometric procedure: In this approach, the density within the detected lumen borders is used to infer lumen dimensions to account for eccentric lesions that may have not been seen in the selected-for-analysis projection (5).

### QCA: reproducibility

Overall, QCA demonstrates good inter- and intraobserver reproducibility. However, several factors can affect its reproducibility, including the size of the guiding catheter used for calibration (17), the selected projections (18), the identification of the end-diastolic frame for analysis, and allowing for the manual contour editing (18, 19). The timing required for QCA analysis is also variable depending on the individual experience of the operator and can be comparable to the time required for intravascular imaging.

QCA analysis in clinical studies is conducted by core labs, which are independent facilities dedicated to delivering unbiased and reproducible results (19, 20). However, they are not immune from inter-core lab variability which is likely the result of differences in the software used for analyses (19, 20), and the standard operating procedures (SOPs) employed (19, 21).

Ideally, angiographic analysis should be paired and matched using the average of multiple matched views whenever possible. In addition, as with any scientific measurement, the analyses should be conducted in a blinded manner to eliminate potential bias from the analysts. However, this can be impractical in certain cases, such as when comparing bioresorbable scaffolds with metallic stents or stenting with balloon angioplasty.

## Clinical implications of QCA

### QCA to guide DES implantation

PCI guided by angiography primarily relies on visual assessment and is subject to high inter- and intraobserver variability. In contrast, QCA provides precise information on vascular dimensions, guiding accurate-sized stents and non-compliant balloons for improving outcomes. The first validation study of QCA using digital computation dates back to 1977 (22). In the 1980s, the introduction of the DICOM system, along with innovative contour detection algorithms, facilitated the measurement of vessels with intricate contours, enhancing QCA-guided PCI. PCI using DES is an established strategy for the treatment of significant obstructive CAD. On-site QCA can be used for optimal stent sizing, ensuring high pressure post-dilatation and optimal stent expansion, and overcoming limitations posed by angiography alone. The Guide DES trial (23) was a randomized investigator-initiated multicenter open-label non-inferiority trial that compared the QCA-guided PCI strategy with intravascular ultrasound (IVUS)-guided PCI in patients with significant coronary artery disease. The trial enrolled 1,528 patients. The Guide DES trial was one of the first studies to compare IVUS with QCA, utilizing an adaptive algorithm designed to align the measurements of IVUS with those of QCA. In this trial, the post-PCI mean (SD) minimum lumen diameter was comparable between the QCA and IVUS groups [2.57 (0.55) vs. 2.60 (0.58) mm, *p* = 0.26]. At 12 months, target lesion failure occurred in 3.81% of the QCA-guided PCI group and 3.80% in the IVUS-guided PCI group [hazard ratio (HR) 1.00; 95% confidence interval (CI), 0.60–1.68; *p* = 0.99]. There were no differences in the rates of stent thrombosis (0.53% vs. 0.66%, *p* = 0.74), coronary perforation (0.2% vs. 0.4%, *p* = 0.41), or stent edge dissection (1.2% vs. 0.7%, *p* = 0.25), between the QCA- and IVUS-guided PCI groups. The occurrence of the primary endpoint remained consistent across subgroups, with no significant interaction observed.

It has previously been demonstrated that QCA underestimates the MLD in small arteries and overestimates the MLD in large arteries compared to IVUS (24). Based on this knowledge, the authors used adjusted QCA to guide stent selection. Adjusted QCA was calculated by adding 10% to the measured reference QCA diameter for vessels ≤3.5 mm, decreasing by 1% for every additional millimeter up to vessels ≥4 mm. Step-wise post-dilatation of the stent with non-compliant balloons was mandated to achieve target diameters, with a recommendation to use stent boost technology when available to ensure optimal expansion. Ultimately, the role of QCA-guided PCI needs to be further determined by meticulously designed clinical trials. That said, the time required to calculate QCA can be influenced by the operator's experience and may, paradoxically, result in longer procedural times compared to intravascular imaging. In addition, adjusted QCA necessitates specialized training for the operator and involves a learning curve.

### Angiography vs. intracoronary imaging-guided PCI

It is important to recognize that GUIDE DES trial enrolled patients with fewer complex lesions [mean SYNTAX score (SS) of 13.3 in the QCA-guided arm and 13.0 in the imaging-guided arm]. Recent studies have shown that lesions at higher risk of stent failure [complex lesions including bifurcation lesions; a chronic total occlusion; unprotected left main (LM) CAD; diffuse lesions with an expected stent length of at least 38 mm; multivessel PCI; use of multiple stents (≥3 planned stents); in-stent restenosis; severely calcified lesion; or ostial lesions of a major coronary] benefit significantly from IVUS imaging or optical coherence tomography (OCT). The RENOVATE-COMPLEX-PCI trial demonstrated that among patients with complex coronary artery lesions, imaging-guided PCI (IVUS or OCT) resulted in a lower risk of a composite outcome, including death from cardiac causes, target vessel-related myocardial infarction (MI), or clinically driven target vessel revascularization, compared to angiography-guided PCI (25). The subanalysis of the ILLUMIEN IV study indicated that in angiographic complex CAD, OCT-guided PCI resulted in a larger minimum stent area (MSA) and reduced serious major adverse cardiac events (MACE)—the composite of cardiac death, target vessel MI, or stent thrombosis—compared to angiography-guided PCI at 2 years; however, it did not significantly improve target vessel failure (TVF) (26). In patients with complex coronary bifurcation lesions, the OCTOBER trial found OCT-guided PCI was linked to a lower incidence of MACE at 2 years compared to angiography-guided PCI (27).

### Acute gain, late loss, and net gain paradigm

**Figure 2:**
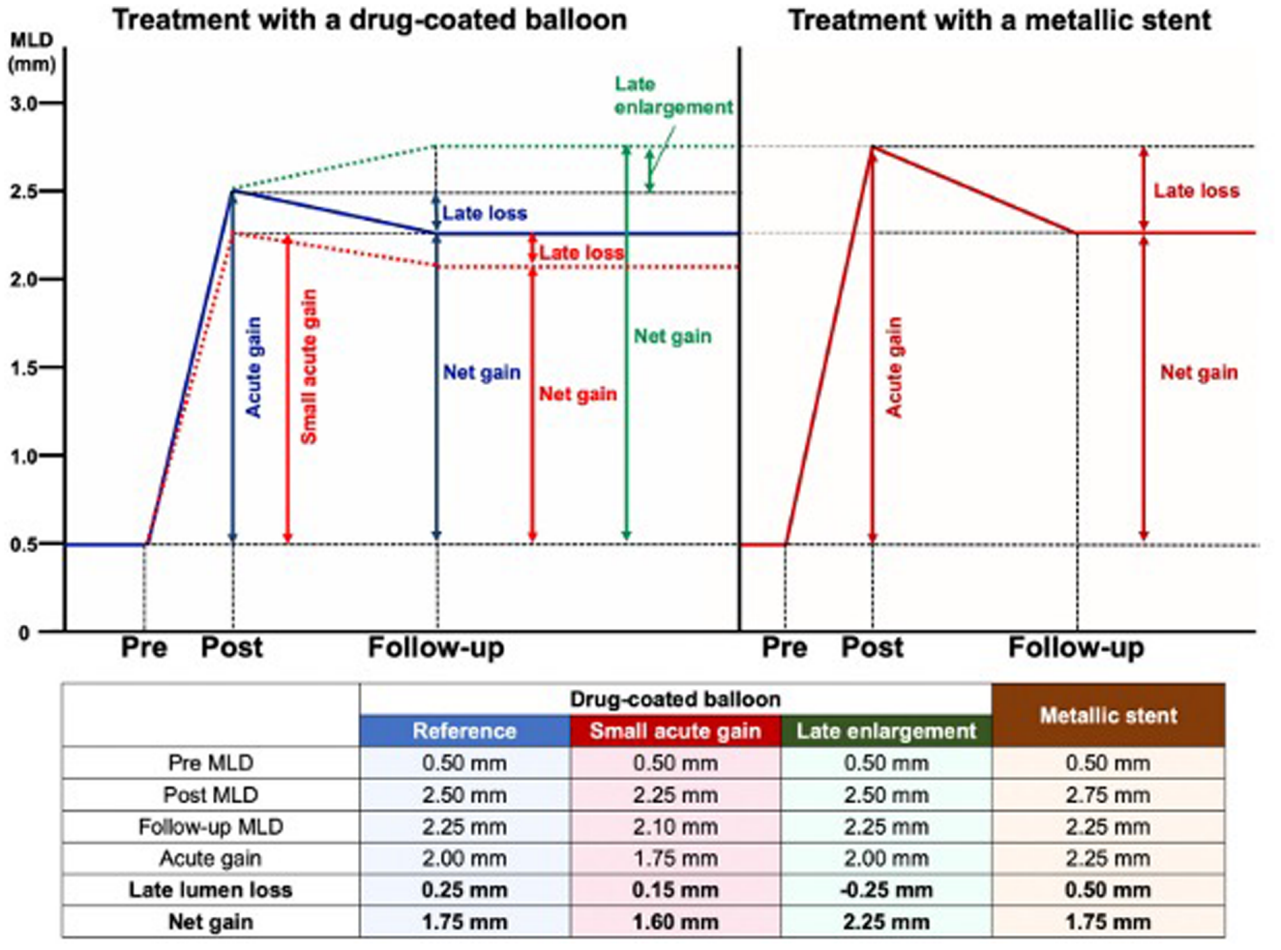
Late lumen loss and follow-up lumen diameter and its implications in clinical trials. The figure illustrates the angiographic changes after treatments with a drug-coated balloon or a metallic stent. Post stenting, there is a predetermined diameter of the metallic cage, implanted with only the biological option to develop intra-stent neointima with reduction of the lumen. However, in the absence of endoluminal prosthesis, late enlargement is feasible and will be accounted for by assessing the net gain (or acute gain + negative late loss). Therefore, net gain is suitable as a surrogate endpoint in DCB studies. DCB, drug-coated balloon; LLL, late lumen loss [Reproduced from Ono et al. (28)].

Understanding coronary restenosis involves grasping the relationship between acute gain, late loss, and net gain. Coronary restenosis is affected by the acute gain from the intervention and the late lumen loss that occurs over the following 4–6 months. Acute gain refers to the increase in luminal diameter from baseline to immediately post-intervention (post-procedure MLD − pre-procedure MLD), while late lumen loss indicates the narrowing from post-intervention to follow-up (post-procedure MLD − follow-up MLD). Consequently, the net gain is the result of the opposing effects of acute gain and late lumen loss (Figure 2). Post-procedural MLD independently predicts both late luminal diameter and percentage of stenosis (29).

The “bigger is better” hypothesis from the early 1990s to explain the benefit of the stent scaffold over angioplasty alone, suggests that, for a similar luminal loss, larger immediate post-procedure MLD leads to a larger late luminal diameter and lower restenosis probability. In the era of the universal use of stents for PCI, where post-procedure MLD varies minimally between stents, late lumen loss became a reliable restenosis metric in pilot and pivotal stent trials. However, late lumen loss is only a reliable comparison metric if acute gain or post-procedure MLD is similar across treatments. Figure 2 illustrates that, despite the lower late lumen loss with balloon angioplasty, the net gain is higher with stenting, resulting in lower restenosis due to greater acute gain. Therefore, in device comparison trials, if acute gain differs (as shown by post-procedure MLD or residual percentage diameter stenosis), late lumen loss reflects neointimal hyperplasia but cannot accurately predict restenosis (30). In drug-eluting vs. bare metal stent trials, the metal scaffolding results in similar acute gain, and hence late lumen loss is a good metric for restenosis probability. This has implications for the design of studies that aim to compare bioabsorbability and durability. It is considered the gold standard for device approval and an important angiographic endpoint for assessing in-stent restenosis (31–34). The LLL is often correlated with neointimal proliferation but ignores constrictive remodeling with balloon angioplasty. For DES, LLL values typically range between 0.1 and 0.3 mm (35, 36). However, LLL as a parameter to assess the neointimal biological reaction has several limitations. First, measurements are taken at two different time points, and each can be influenced by systematic and random errors. Second, a focal measurement does not represent the biological processes occurring along the entire length of the stent. Third, the location of the smallest diameter immediately after the procedure may shift along the stent length at follow-up, potentially resulting in discrepancies between LLL and neointimal volume (37). A patient-level meta-analysis of seven randomized controlled trials (RCTs) conducted by Asano et al. examined the effect of angiographic late LLL on the incidence of long-term target lesion revascularization (TLR) and identified a specific threshold that influences events through pooled patient-level and study-level analyses. This study revealed an exponential relationship between in-stent LLL and the incidence of TLR, indicating that patients with LLL greater than 0.50 mm are likely to require TLR during long-term follow-up. However, low LLL values may not effectively predict TLR. The optimal cut-off value for predicting TLR with acceptable sensitivity and specificity was determined to be 0.50 mm. This threshold can be utilized in clinical decision-making and in establishing a non-inferiority boundary for efficacy endpoints (38) (Supplementary Figure S1).

## QCA assessment in bifurcations

One of the major limitations of conventional QCA is its efficacy in assessing bifurcation lesions. Understanding coronary fractal geometry is crucial for the application of QCA software in this setting. Various scaling laws have been proposed, highlighting that the distal vessel diameter is always smaller than the proximal vessel due to step-down change in reference diameter at the site of the bifurcation. The most accurate and widely accepted is the Huo–Kassab model (16, 39, 40). The Huo–Kassab model provides a mathematical framework for understanding the fractal nature of the coronary vasculature, allowing for more accurate measurements and assessments in complex coronary anatomy, including bifurcations.

According to this model, the proximal main vessel (PMV) (mother) and distal main vessel (DMV) (daughter) hold the following relationship: *D_m_*^7/3^ = *D*_l_^7/3^ + *D_s_*^7/3^, where *m*, *l*, and *s* represent the mother, larger, and smaller daughter branches, respectively.

**Figure 3:**
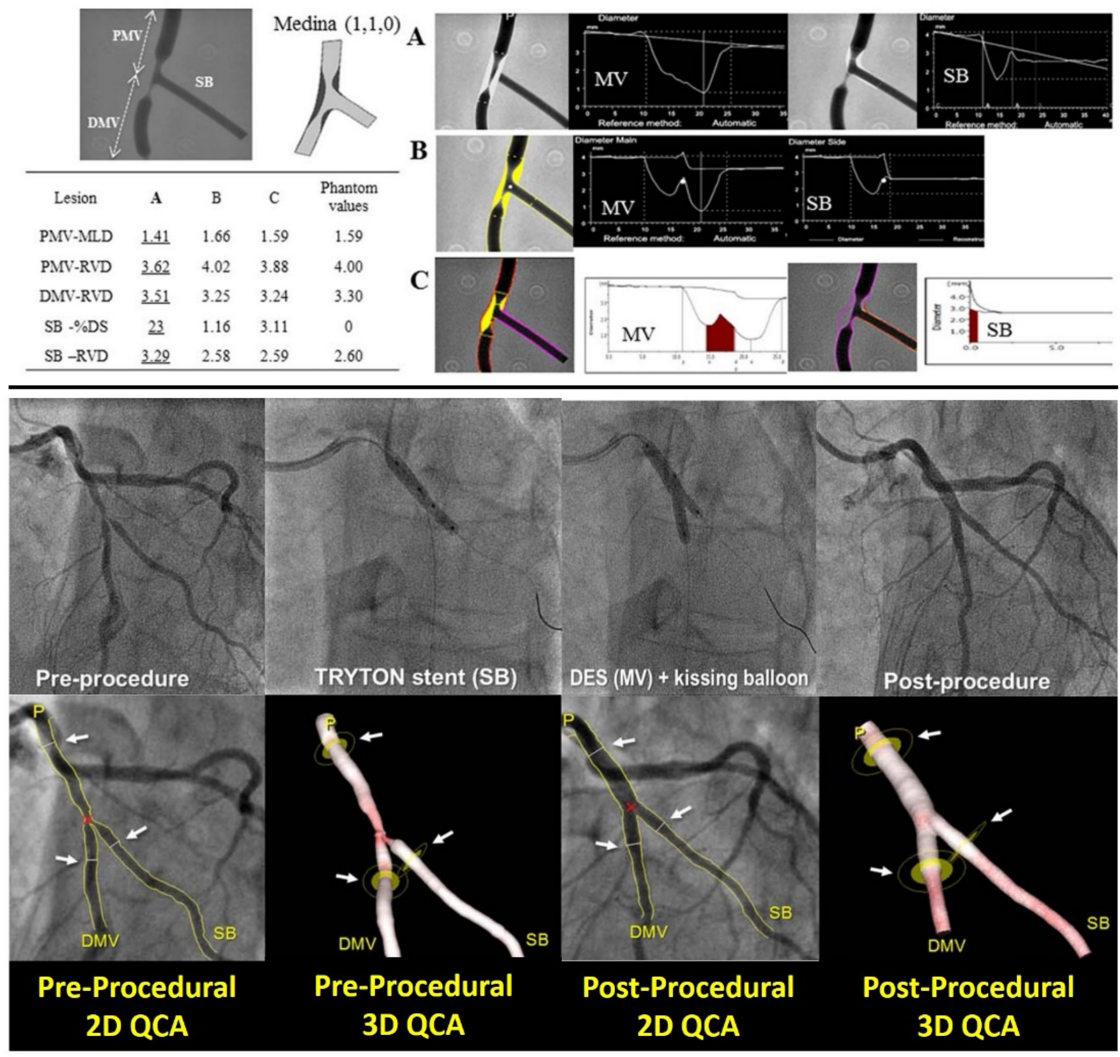
Comparison of different software algorithms for coronary bifurcation analysis using calibrated phantoms (upper panel) and comparison between 2D and 3D quantitative coronary angiography bifurcation analyses (lower panel). Upper panel: This is an example of a case of the phantom model results. Quantitative coronary angiography measurement by CAAS and QAngio XA with automatic selection of the Y- or T-shape algorithm is shown for a bifurcated lesion in the calibrated phantom. Angiographic parameters including RVD, minimal lumen diameter, % diameter stenosis are given in the PMV, DMV, and SB. Case 1: A cine-angiogram demonstrated moderate to severe stenosis both in the PMV and the DMV in the Medina class (1,1,0) bifurcation. The conventional single-vessel method measured a significantly smaller RVD in the PMV and a larger RVD in the DMV and SB. **(A)** QCA is shown using the conventional single-vessel algorithm by CAAS. **(B)** QCA is shown using the bifurcation algorithm by CAAS. **(C)** QCA is shown using the bifurcation algorithm by QAngio XA with automatic selection of Y or T shape [Reproduced from Ishibashi et al. (41)]. Lower panel: Treatment procedure using the Tryton stent and the definition of the treated segment. A bifurcation lesion was observed in the mid segment of left anterior descending artery and a diagonal branch [left in **(A)**]. After pre-dilatation, a Tryton stent was implanted toward the side branch (center left), then a drug-eluting stent was implanted through the Tryton stent in the main vessel (center right). The final angiogram showed good results (right). The treated segments were delineated using three white lines (see white arrows) at the proximal main branch (PMB), distal main branch (DMB), and SB in the matched projections [white arrows, pre-procedure in **(B)** and post-procedure in panel **(C)**]. Specifically, the proximal and distal borders of the main vessel were set at the proximal and distal edge of the DES implanted, respectively. In this case, the distal border of side branch was defined as the distal edge of the Tryton stent [Reproduced from Muramatsu et al. (42)].

Single-vessel QCA software ignores this relationship in bifurcating vessels, resulting in dubious results. Specifically, single-vessel QCA in bifurcation lesions tends to underestimate the percentage of diameter stenosis in the mother vessel and overestimate its severity in the distal main and side branches. To overcome these limitations, dedicated bifurcation QCA software was developed (Figure 3).

## QCA using dedicated QCA bifurcation software

The bifurcation segmentation is initiated by placing one proximal and two distal delimiter points at the maximum possible distance from the bifurcation to be analyzed. These points can be adjusted as needed by the analyst (43).

The software utilizes various anatomical points to define the components of the bifurcation and their measurements. The CAAS bifurcation QCA software employs the point of bifurcation (POB) and the polygons of confluence (POC) to calculate the diameter of each bifurcation component, applying different algorithms for areas inside and outside the POC (43, 44).

In contrast, the QAngio XA software uses the carina point on the middle contour as the foundation for defining the four “building blocks” of the bifurcation analysis model: PMV, bifurcation core, DMV, and SB. Regardless of the software utilized, to achieve QCA analyses, operators, the designated core laboratory, or site analysts should adhere to the following guidelines (45):

(1) For optimal visualization of the lesion, two angiographic projections orthogonal to the bifurcation plane should be obtained. These projections must be separated by at least 30° to enable dedicated QCA bifurcation analysis. The quantitative analysis should be conducted in two views that do not exhibit vessel overlap or minimal foreshortening and showcase the widest bifurcation angle.
(2) A qualitative assessment of the bifurcation lesion, including factors such as calcification and the presence of thrombus, should be documented for each of the three segments.
(3) Bifurcation angles should be reported before the intervention, after the intervention, and at follow-up. The angle between the PMV and the SB is referred to as Angle A (Access), which affects the accessibility of the SB for stenting. Angle B (Between) represents the angle between the two distal branches and indicates one of the risks for SB occlusion during main branch (MB) stenting, while Angle C is the angle between the PMV and the DMV (46). Importantly, vessel angulation and tortuosity may hinder the ability to obtain the aforementioned projections.
(4) The assessment of bifurcation lesion dimensions, severity, and extension should be conducted using a segmental analysis. MLD, reference vessel diameter (RVD), and %DS should be reported for each coronary segment, including the PMV, DMV, and SB. For post-procedural and follow-up analyses, it is recommended to report these three measurements for each component of the six-segment model (BSM6): PMV = segment 2; DMV = segment 3; SB = segment 5; 5 mm segment beyond the treated PMV segment = segment 1; 5 mm segment beyond the treated DMV segment = segment 4; and 5 mm segment beyond the treated SB segment = segment 6. Segments 2, 3, and 5 are divided by the POB. In addition, an 11-segment model (BSM11) analysis may be reported as follows: POC = segment 7; 3 mm ostial segment of the SB = segment 8; DMV = segment 11; entire main vessel = segment 9 (segments 1, 2, 3, and 4); and the entire SB = segment 10 (segments 5 and 6). The BSM11 model provides a more detailed definition of specific bifurcation portions, such as the SB ostium; however, it requires longer reporting times and, due to its increased complexity, is more susceptible to analysis-dependent errors.
(5) The same segmental analysis employed during post-intervention should be applied to follow-up analyses. This segmental analysis will offer a detailed analysis of the location of any residual stenosis after the intervention and pinpoint the exact site of treatment failure or restenosis during follow-up.
(6) The size of the SB should be defined as the RVD at the ostium of the SB, specifically the 3 mm segment from the POC contour and before any secondary bifurcation. This corresponds to the 3 mm proximal portion of segment 5 in the six-segment model (BSM6) and to segment 8 in the eleven-segment model (BSM11).
(7) The highest %DS and the MLD should be reported as a single metric for the entire bifurcation lesion.

To address potential limitations associated with 2D-QCA of bifurcation lesions, such as vessel overlap, tortuosity, and foreshortening, dedicated 3D-QCA software packages have been developed. These include the CAAS QCA 3D system (Pie Medical Imaging, Maastricht, the Netherlands) and QAngio XA 3D (Medis Medical Imaging Systems, Leiden, the Netherlands) (47, 48). In these software packages, a 3D coronary reconstruction is created from two 2D image datasets, and specialized QCA algorithms for bifurcation lesions are utilized for automatic calculations (49, 50). In a study conducted by Tu et al., fractional flow reserve (FFR)_3D-QCA_ was calculated for 77 vessels across 68 patients. The average diameter stenosis was 46.6 ± 7.3%. FFR_QCA_ strongly correlated with FFR (*r* = 0.81, *p* < 0.001). The area under the receiver-operating characteristic (ROC) curve was 0.65 for percentage diameter stenosis, 0.73 for minimal lumen area, and 0.93 for FFR_QCA_ (51). Moreover, a 3D reconstruction of the bifurcation enables a more precise measurement of the bifurcation angle compared to 2D-QCA. Since the bifurcation is a 3D structure, its maximal opening can only be accurately appreciated in three dimensions (47), a finding supported by a phantom study (49). The significance of accurately measuring bifurcation angles arises from previous studies exploring their relationship with clinical outcomes, although the evidence remains inconsistent. For instance, Watanabe et al. (52) demonstrated that a large pre-stent systolic–diastolic distal LM bifurcation angle (Angle B) greater than 7.2° is associated with a fivefold increase in the risk of target lesion failure at 3 years. In contrast, the SYNTAX trial found that the pre-procedural diastolic angle did not affect outcomes, while a restricted post-procedural systolic–diastolic Angle B of less than 10°, which indicates bifurcation stiffening and altered shear stress, was linked to higher 5-year adverse event rates following a LM bifurcation PCI (53). In addition, data regarding the risk of SB occlusion related to Angle B are also contradictory, with some studies identifying an acute Angle B as a predictor of SB impairment (54), while others report the opposite (55).

## Three-dimensional QCA: does it give true perspective?

Three-dimensional imaging has recently surpassed two-dimensional imaging, enhancing spatial orientation and improving our coronary artery reconstruction. 3D-QCA requires images captured at two projection angles separated by ≥30°. The major advantage of 3D-QCA lies in its precision in estimating length, reducing possible foreshortening (42, 47, 49). Furthermore, it excels in accurately measuring the MLD in eccentric lesions. Given the oval shape of their lumens, the MLD varies with the projection angle; when the viewing angle aligns perpendicularly to the shortest axis of the oval-shaped lumen, the MLD is the smallest. 3D-QCA demonstrates superior predictability for functional significance compared to 2D-QCA (56).

3D-QCA can calculate the optimal viewing angle, defined as an orthogonal view of the lesion, overcoming foreshortening and overlapping (57). In addition, it facilitates 3D modeling using computational fluid dynamics (CFD), allowing for physiological assessments without the need for vessel instrumentation with pressure and/or flow wires (58, 59).

However, while 3D-QCA provides precise measurements of the lumen dimensions, it is unable to assess the vessel wall and detect early plaque formation and vessel wall remodeling (60). Furthermore, intrinsic angle limitations of the C-arm of angiographic systems preclude making an accurate 3D reconstruction of bifurcations in critical locations, such as the left main coronary vessel, in many cases. Techniques to address these limitations are discussed below.

## QCA in comparison with IVUS/OCT

High-resolution intravascular imaging techniques, such as IVUS and OCT, have significantly enhanced our understanding of atherosclerosis pathology and vessel response following stent implantation. These modalities allow for a detailed assessment of the plaque burden and distribution, making intravascular imaging superior to traditional QCA for precise plaque quantification. The above qualities of IVUS and OCT have been useful in PCI planning, especially for complex lesions such as chronic total occlusions, long lesions, left main stem disease, and bifurcation lesions. The ability to visualize vessel walls enables accurate stent sizing and the identification of the landing zones with minimal plaque burden, reducing the risk of geometric miss. They can also be used to detect common causes of stent failure post-PCI such as under expansion, major edge dissections, geographic miss, and thrombus protrusion (61).

### 2D-QCA vs. 3D-QCA vs. intravascular imaging

Tsuchida et al. developed a new 3D visualization and quantitative analysis software system (CardiOp-B™), which was validated in *in vivo* experimental settings against both 2D and 3D-QCA (24). Using OCT, phantom lumen diameters were also evaluated *ex vivo*. Precision-drilled plexiglass phantoms with five different lumen diameters, ranging from 0.5 to 1.9 mm, were percutaneously inserted into the coronaries of four Yorkshire pigs. A total of 22 angiographic images of the artificial phantom coronary stenoses in the pigs were acquired as part of the *in vivo* validation test. Quantitative assessments of the minimum and mean lumen diameters were conducted using both QCA systems, while *ex vivo* images of the same phantom lumens were captured and measured using OCT. The study found that the accuracy of luminal diameter measurements was superior with the current 2D-QCA system compared to the 3D-QCA systems. However, OCT yielded excellent results, demonstrating precise phantom diameter measurements. In the SPIRIT FIRST study, Tsuchida et al. (62) examined 56 in-stent segments (27 with everolimus-eluting stents and 29 with bare metal stents) to compare QCA measurements with corresponding IVUS parameters. Two IVUS-late loss models were derived from the MLD using either a circular model or a projected MLD. QCA-neointimal volume was calculated by subtracting the lumen volume (mean area of the stented segment × stent length) at follow-up from the stent volume (mean area of the stented segment × stent length) post-procedure, with the stent length determined either from nominal stent length or measured by QCA. In addition, the videodensitometric neointimal volume was evaluated. Each of the three neointimal volume measurements and the percentage volume obstruction obtained by QCA demonstrated a significant correlation with the corresponding IVUS parameters (*r* = 0.557–0.594, *p* < 0.0001), although a broad range of limits of agreement was noted. Furthermore, late loss and volumetric measurements obtained by QCA exhibited a wider range of standard deviations compared to those measured by IVUS.

### Multislice computed tomography vs. QCA

A study conducted by Bruining et al. (63) aimed to assess whether 3D quantitative techniques are comparable to the standard 2D-QCA method and to evaluate the feasibility of using non-invasive multislice computed tomography (MSCT) for quantifying luminal dimensions in a stented coronary segment with a novel bioabsorbable drug-eluting stent made of poly-L-lactic acid (PLLA). The results demonstrated that 3D-based quantitative analyses yielded similar outcomes to 2D-QCA in measuring luminal dimensions after PCI with the bioabsorbable coronary stent design. Furthermore, the study indicated that non-invasive QMSCT-CA could effectively quantify luminal dimensions in cases involving biodegradable PLLA scaffolds. However, it is crucial to recognize that reconstructing the arterial tree with MSCT eliminates the limitations associated with invasive angiography, which is constrained by the limited number of angulations provided by the C-arm.

### QCA vs. MSCT vs. intravascular imaging

**Figure 4:**
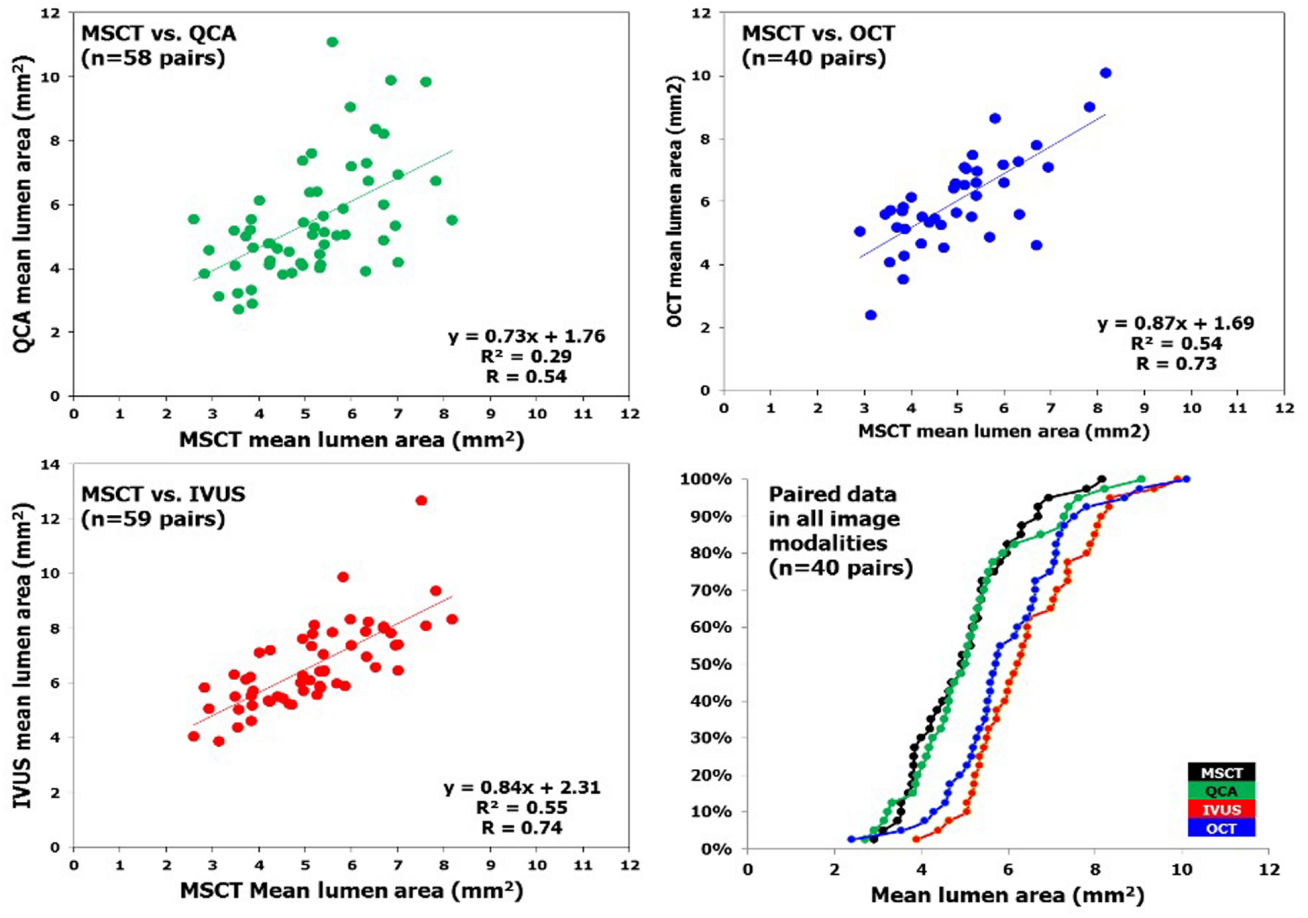
In patients who underwent bioresorbable vascular scaffold implantation, correlation of mean lumen area measured by MSCT and invasive coronary imaging at long-term follow-up is illustrated. Linear regression of MLA measured by multislice computed tomography (*x* axis) and the different imaging modalities (*y* axis). The right lower panel depicts the cumulative curve of mean lumen area measured by different imaging techniques. IVUS, intravascular ultrasound; MSCT, multislice computed tomography; OCT, optical coherence tomography; QCA, quantitative coronary angiography [Reproduced from Suwannasom et al. (64)].

In a study examining the fate of metallic radio-opaque markers (MRM) from bioresorbable vascular scaffolds (BVS) at the implantation site post-bioresorption, a total of 168 lesions were analyzed using MSCT at 18 months. This included 12 lesions from ABSORB Cohort A, 61 lesions from ABSORB Cohort B, and 95 lesions from the ABSORB EXTEND study. A paired comparison for lumen area was done between MSCT and QCA, MSCT and OCT, and MSCT and IVUS. A total of 348 MRMs were assessed through both quantitative and qualitative analyses; all MRMs were located at the implantation site, with no signs of embolization to distal vascular beds. The median scaffold length measured by MSCT was 18.0 mm, with a range from 12 to 36 mm, and was identical to the median nominal scaffold length (18.0 mm, ranging from 12 to 28 mm). Thus, the median length difference between the MSCT and nominal scaffold lengths was 0.0 mm (IQR: 1.0–1.0 mm). A moderate correlation was found between the mean lumen area (mean LA) measured by MSCT and that measured by QCA (*r* = 0.54, *p* < 0.0001). In addition, strong correlations were observed between MSCT mean LA and both IVUS mean LA and OCT mean LA (*r* = 0.74 and 0.73, respectively; *p* < 0.0001). While the mean LA from MSCT was comparable to that from QCA, it was statistically lower than those obtained from IVUS and OCT. The reproducibility of the four criteria for identifying MRMs from calcified nodules (CN) was excellent (*r* = 0.97; *p* < 0.0001). (64) (Figure 4).

### Artificial intelligence-based coronary stenosis quantification by coronary CT angiography vs. QCA and IVUS

Machine learning methods for quantifying anatomic stenosis are increasingly being explored to enhance interpretation efficiency and boost reader confidence. A recent multicenter study utilizing an artificial intelligence (AI)-based anatomical CT tool, with QCA serving as the reference standard, showed high diagnostic performance at both 50% and 70% stenosis thresholds when compared to QCA (65). The AI-based tool demonstrated strong discriminatory capability for anatomic stenosis, showing high sensitivity and negative predictive value, which underscores its clinical significance. This *post hoc* analysis involved 120 participants (mean age: 59.7 years, 60.8% men) drawn from three major clinical trials (AFFECTS, P3, REFINE) who underwent coronary computed tomography angiography (CCTA) and invasive coronary angiography (ICA) with QCA. An AI-based coronary stenosis quantification (AI-CSQ) software was used for the quantitative analysis of coronary stenosis severity in CCTA. A blinded comparison between QCA and AI-CSQ was conducted on both a per-vessel and per-patient basis. The AI-CSQ tool demonstrated strong diagnostic performance for identifying DS. For DS of 50% or greater, it achieved a sensitivity of 80%, specificity of 88%, accuracy of 86%, positive predictive value of 65%, and negative predictive value of 94%. For DS of 70% or greater, the corresponding values were 78% sensitivity, 92% specificity, 91% accuracy, 47% positive predictive value, and 98% negative predictive value. The areas under the ROC curve (AUC) were 0.92 and 0.93 for predicting DS of 50% and 70%, respectively, on a per-vessel basis. On a per-patient basis, the AUCs were 0.93 for DS of 50% and 0.88 for DS of 70%. All results were statistically significant (*p* < 0.001).

A closely related investigation, the pRospEctive multicEnter study to AnaLyze PLAQUE (REVEALPLAQUE) study (66), assessed the level of concordance between AI-enabled quantitative coronary plaque analysis (AI-QCPA) and IVUS. This prospective blinded core lab-adjudicated multinational study exhibited strong agreement with the current IVUS standards concerning lumen, plaque burden, and morphology. The correlation coefficients for total plaque volume, calcified plaque volume, and non-calcified plaque volume were 0.91, 0.91, and 0.87, respectively. Bland–Altman analysis indicated strong agreement with minimal bias for these measurements.

### Quantitative multi-modality imaging analysis of a fully bioresorbable scaffold: QCA vs. IVUS vs. OCT

In DESs, QCA using video densitometry often overestimates the minimal lumen area (MLA) because of the radiodensity of the metallic struts. However, BVS can potentially address this issue, as they are translucent to optical radiation and radiolucent to gamma radiation, except for the radiopaque platinum markers located at their edges. In a study by Gutiérrez-Chico et al. (67), 45 patients from the ABSORB cohort B1 underwent coronary angiography, IVUS, and OCT immediately after BVS implantation and at 6 months follow-up. OCT accurately estimated stent length compared to nominal length, with a 95% confidence interval for the difference of −0.19 to 0.37 mm at baseline and −0.15 to 0.47 mm at 6 months. In contrast, QCA consistently underestimated stent length at both time points. IVUS yielded low accuracy, with several outliers and random variability in test-retest at baseline and 6 months follow-up. The MLA decreased significantly on QCA and OCT between baseline and 6 months, but only minimally on IVUS (95% CI: 0.11–0.42). The agreement among imaging modalities was poor, with the worst agreement observed between videodensitometry and IVUS post-implantation (ICC *a* = 0.289) and the best agreement between IVUS and OCT at baseline (ICC *a* = 0.767). All comparisons deviated significantly from linearity (*p* < 0.01). Overall, OCT proved to be the most accurate method for measuring stent length, while QCA faced systematic underestimation (foreshortening) and solid-state IVUS exhibited random error. Therefore, volumetric calculations using solid-state IVUS following BVS implantation were unreliable, and there was poor agreement for MLA estimation among all the studied imaging modalities, indicating that their values are not interchangeable.

## Anatomical SYNTAX score

The anatomical SYNTAX score (aSS) has emerged as an anatomical tool to objectively determine the complexity of CAD and to guide decision-making between PCI and coronary artery bypass grafting (CABG) (68–70). There have been numerous validation studies, including the landmark SYNTAX trial comparing CABG vs. PCI in patients with complex CAD, confirming the efficacy of the score to detect higher-risk subjects and aid decision-making (70, 71). Furthermore, the U.S. Food and Drug Administration has mandated the SYNTAX score as entry criteria in the major contemporary stent and ongoing structural heart disease trials, for example, in the EXCEL trial (Evaluation of XIENCE PRIME or XIENCE V Everolimus-Eluting Stent System Versus Coronary Artery Bypass Surgery for Effectiveness of Left Main Revascularization) and SURTAVI trial (Safety and Efficacy study of the Medtronic CoreValve System in the Treatment of Severe, Symptomatic Aortic Stenosis in Intermediate-Risk Subjects Who Need Aortic Valve Replacement).

**Figure 5:**
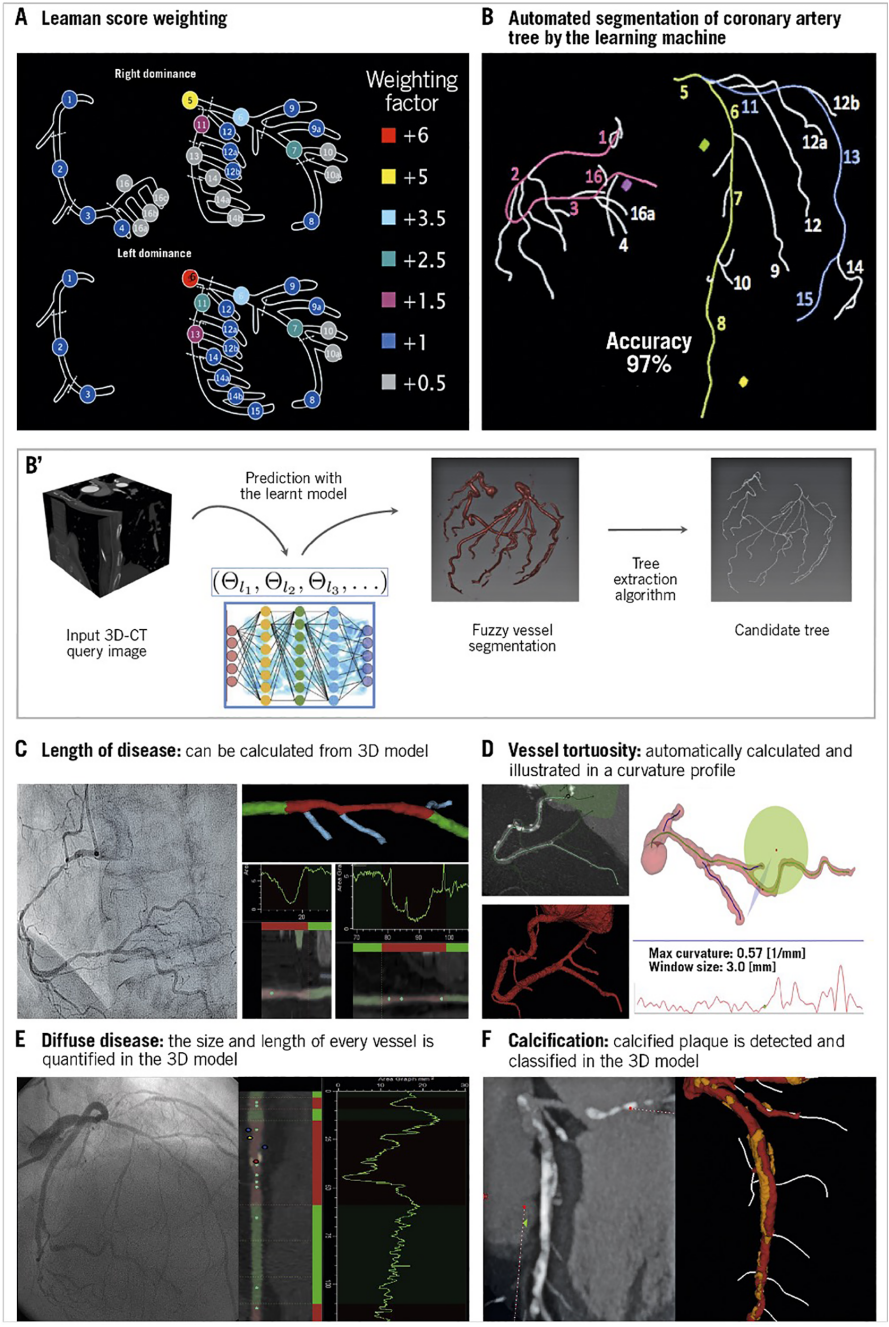
Anatomic SYNTAX score evaluated from the multislice CT scan with automated segmentation produced by the learning machine: **(A)** Leaman score weighting, **(B,B')** automated segmentation of coronary artery tree by machine learning, **(C)** calculating the length of the lesion, **(D)** estimation of vessel tortuosity, **(E)** estimation of diffuse disease, and **(F)** estimation of calcification [Reproduced from Serruys et al. (76)].

The anatomical SYNTAX score was born during the design of the SYNTAX trial as a tool to systematically analyze coronary angiograms and to specify the number of coronary lesions that require treatment and assess their anatomical location and complexity (68–71). The SYNTAX score combines the importance of a diseased coronary artery segment in terms of its severity (i.e., obstructive or occlusive), anatomic location, its implications on the myocardial blood supply (vessel segment weighting based on the Leaman Score) (72), and adverse lesion characteristics [American College of Cardiology (ACC)/American Heart Association (AHA) lesion classification (73) and Medina classification (74, 75)]. Each vessel segment 1.5 mm in diameter or greater (Figure 5), labeled 1 through 16, with a 50% or more diameter stenosis by visual estimation is given a multiplication factor related to the coronary lesion location and severity (see Figure 5A). Further characterization of the coronary lesions leads to the addition of more points (see Figure 5B) and includes features of total occlusions (duration, length, blunt stumps, and presence of bridging collaterals or side branch), bifurcation (Medina classification) or trifurcation (number of diseased branches involved), side branch angulation, aorto-ostial lesions, severe tortuosity, lesion length greater than 20 mm, heavy calcification, thrombus, and diffuse or small-vessel disease. An online SYNTAX score algorithm (77) automatically summates each of these features to calculate the total SYNTAX score.

Within the SYNTAX trial (78), the distribution of the SYNTAX score was found to be normal (Gaussian) in the randomized PCI and CABG populations with the curves almost being superimposable on each other. When the scores of the randomized SYNTAX population were divided into tertiles, the upper boundary of the lowest tertile was 22 (low risk), the second tertile ranged from 23 to 32 (intermediate risk), and the lower boundary for the highest tertile was equal to or greater than 33 (high risk).

Based primarily on the results of the SYNTAX trial (78–80), current European revascularization guidelines (81) give to subjects with three-vessel disease (3VD) and low SYNTAX scores (0–22) without diabetes mellitus a class I recommendation, level of evidence (LOE) A, for both PCI and CABG. Furthermore, the guidelines give subjects with 3VD and low SYNTAX scores (0–22) with diabetes mellitus a class I recommendation, a LOE A, for CABG, and a class IIb LOE A recommendation for PCI. In subjects with unprotected left main coronary artery (ULMCA) disease and low to intermediate SYNTAX scores (<33), a class I LOE A recommendation is given for CABG, a class I LOE A for PCI when their SYNTAX score is low (0–22), and a class II LOE A for PCI when their SYNTAX score is intermediate (23–32) (81). Furthermore, U.S. guidelines currently give surgical revascularization for ULMCA disease a class I B recommendation (82) compared with a class I A recommendation in previous guidelines (83).

Anatomical SYNTAX score can now be evaluated from a multislice CT scan with automated segmentation produced by artificial intelligence (Figure 5).

**Figure 6:**
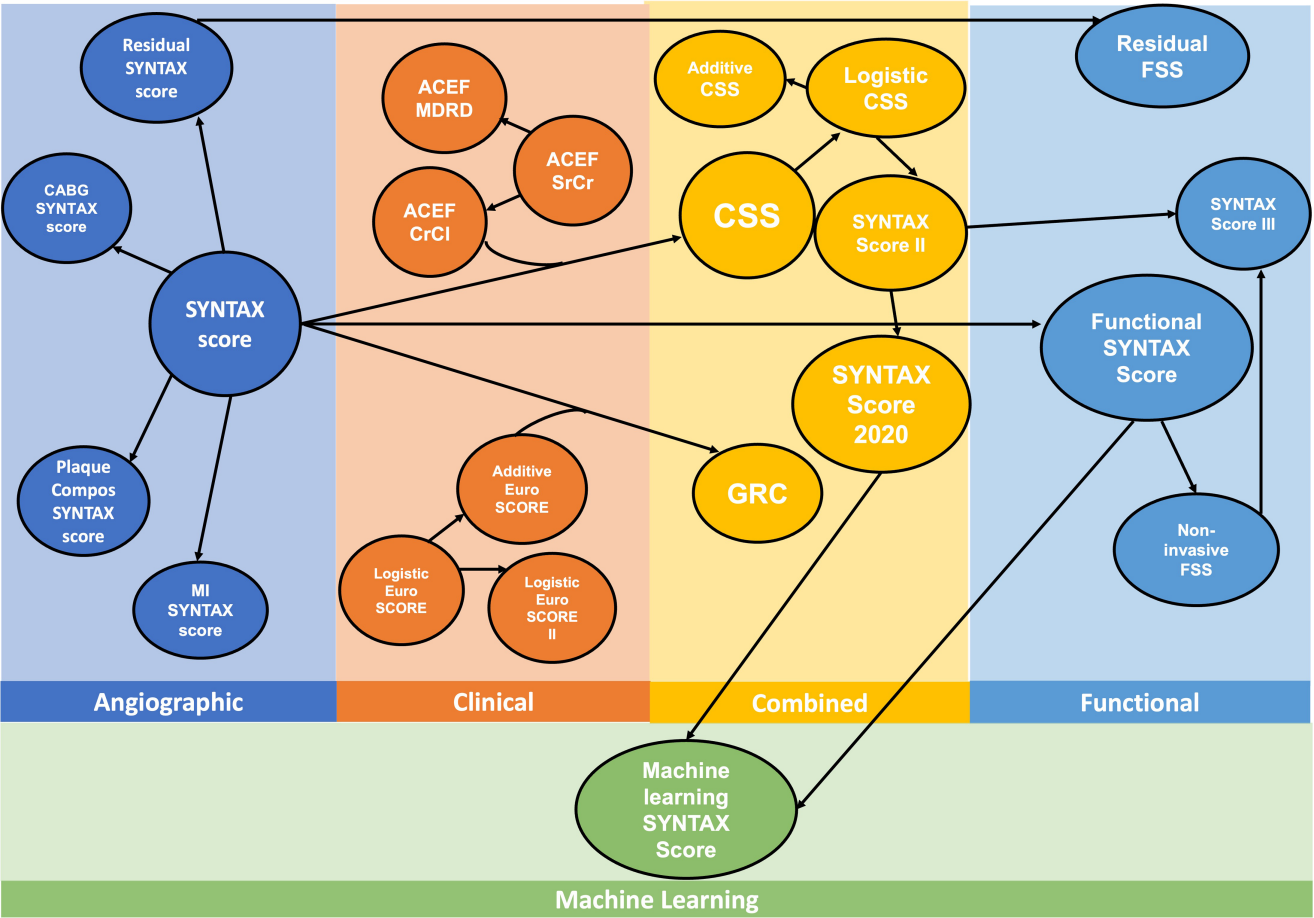
Evolution of the risk score algorithms derived from the historical SYNTAX I trial. ACEF, age, creatinine, ejection fraction; CABG, coronary artery bypass grafting; Compos, compositional; CrCl, creatinine clearance; CSS, clinical SYNTAX score; FSS, functional SYNTAX score; GRC, global risk classification; MI, myocardial infarction; MDRD, Modification of Diet in Renal Disease; SrCr, serum creatinine; SYNTAX, SYNergy between PCI with TAXus and Cardiac Surgery [Modified from Serruys et al. (84)].

Anatomical SYNTAX score has been an integral part of risk scores, and prediction models have been developed over time based on the data of the SYNTAX study, which is summarized in Figure 6.

## Transition from QCA to angio-based FFR: functional coronary angiography

Epicardial stenosis in coronary arterial disease is identified by invasive coronary angiography. Coronary artery stenosis with diameter stenosis >50% is considered obstructive, as the coronary flow reserve (CFR) would be declined (85). In the 1970s, Gould and his colleagues identified a gap between the geometry and functional relevance of coronary stenosis. Coronary angiography can lead to an over or underestimation of lesion severity (86). This led to the emergence first of CFR and, subsequently, of FFR, which could estimate the functional stenosis severity of CAD and thus guide revascularization better than CFR (87, 88). Thus, many authors began referring to FFR as the new gold standard. Several studies reported a functional mismatch between QCA-DS and FFR estimations in one-third of the patients with intermediate stenosis (86). Another study using IVUS, highlighted the difference between QCA-DS and FFR and attributed it to factors such as MLD, lumen length, plaque burden, and the presence/absence of plaque rupture (89). Another group showed that these differences are also affected by the presence of microvascular function (90), and the myocardial mass subtended by the lesion (91). This implies that QCA-DS limited decision-making and can lead to the revascularization of functionally non-significant lesions. When it comes to assessing and guiding percutaneous treatment of intermediate coronary lesions in chronic coronary syndromes (CCS), invasive pressure wire (PW) derived fractional flow reserve (PW-FFR) and the instantaneous wave-free ratio (PW-iFR) share the gold standard status, and have class I and IIa recommendations in European and U.S revascularization guidelines, respectively (81, 82).

Despite the above recommendation, there is limited utilization of PW-FFR/PW-iFR in routine clinical practice. This is attributed to multiple factors, including the need for intracoronary instrumentation with harder-to-manipulate wires and related procedural complications, the need to induce hyperemia adding to patient discomfort and increasing procedural time, and because of variable reimbursement.

Recently, several novel wireless technologies have been developed that incorporate computational fluid dynamics to predict pressure drop across lesions.

**Figure 7:**
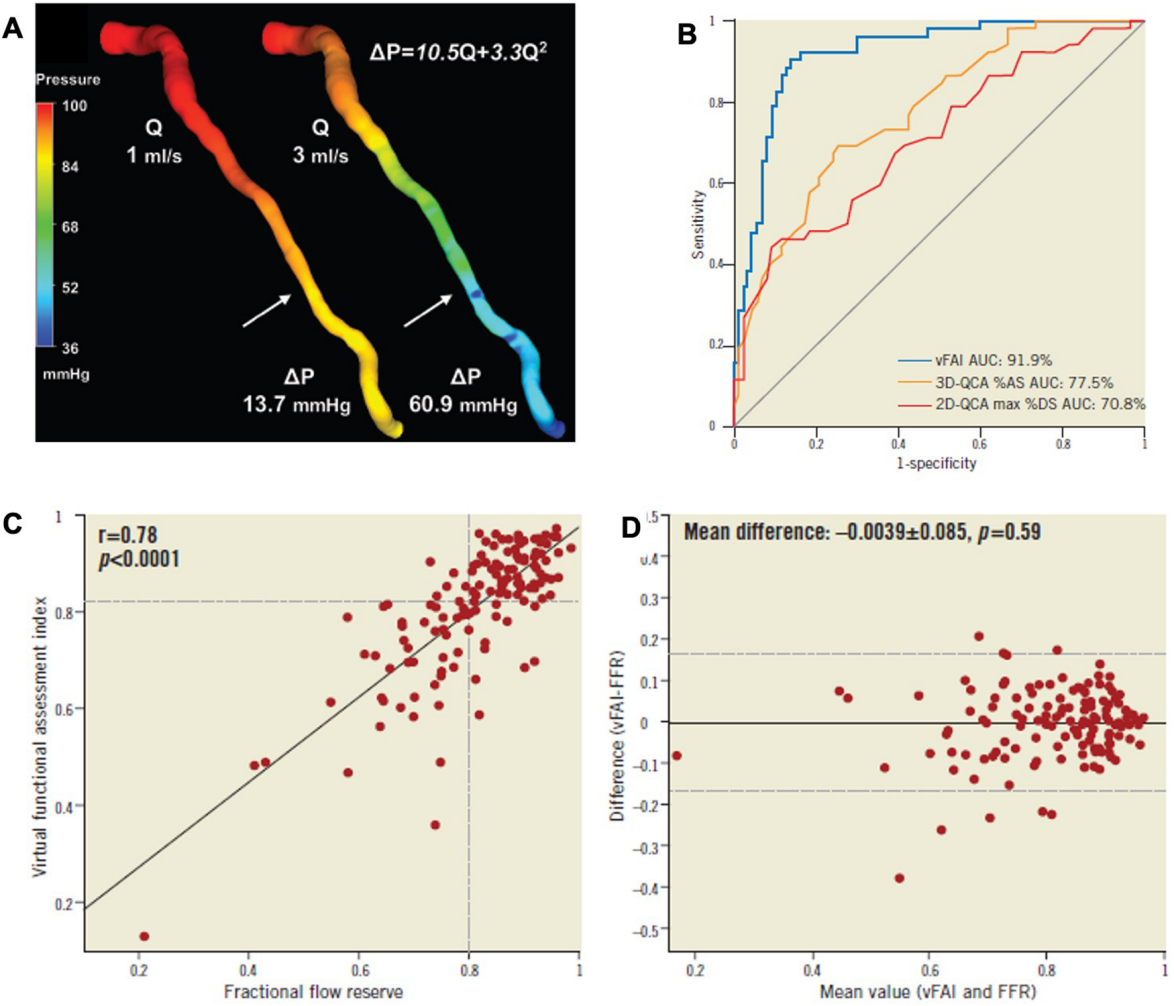
Functional assessment of intermediate coronary lesions using quantitative coronary angiography. **(A)** 3D-QCA of an LAD artery with a moderate lesion (arrow: maximal stenosis) in angiography [3D-QCA %diameter stenosis (%DS): 35%] that had a low fractional flow reserve (FFR = 0.64) measured at a distal location (dotted arrow) using the pressure wire coronary lumen reconstruction with the pressure distribution in a color-coded map for two different flow rates (*Q*), which resulted in a pressure gradient (Δ*P*) of 13.7 and 60.9 mmHg. The computed artery-specific Δ*P*-*Q* relationship is provided. The arrows denote the location of maximal stenosis. **(B)** The AUC was 91.9% (95% CI: 86%–96%). The respective AUC for 3D-QCA %area stenosis [%AS; AUC: 77.5% (95% CI: 69.9%–84.3%); values on the ROC curve represent 1 − AS) and 2D-QCA max %DS [AUC: 70.8% [95% CI: 62.2%-77.9%]; values on the ROC curve represent 1 − DS) showed that the virtual functional assessment index (vFAI) had significantly higher discriminatory power (*p* < 0.001 for both). **(C,D)** Relationship between the ratio of distal to aortic pressure (Pd/Pa) and flow for the studied artery, and calculation of the artery-specific vFAI (0.62) shows the good agreement and correlation with wire-FFR. ROC curve analysis for the vFAI against the fractional flow reserve cut-point (≤0.80: reference standard) [Reproduced from Papafaklis et al. (92)].

Quantitative flow ratio (QFR) is calculated using fluid dynamic principles and three-dimensional angiography, producing a virtual, color-coded display of FFR values on 3D-QCA without requiring a pressure wire or inducing hyperemia. The pressure loss across a coronary stenosis (Δ*P*) is influenced by the severity of the narrowing and the magnitude of flow (*Q*) passing through it. This pressure loss results from two factors: (1) viscous friction (*f*) and (2) flow separation due to acceleration through the stenosis (*t*), which creates swirling blood flow and reverse currents. The equation *ΔP* = *fQ*^2^ + *tQ*^2^ illustrates that pressure loss through a stenosis increases quadratically with rising coronary flow. To obtain patient-specific estimates of blood flow and pressure in coronary arteries based on coronary angiography, four essential steps must be undertaken: (1) choose a fluid equation solver (either computational fluid dynamics or simplified fluid dynamics equations), (2) reconstruct a 3D model of the coronary arteries, (3) define boundary conditions, and (4) specify flow velocity (Figure 7).

There have been several studies demonstrating a good correlation between angio-based FFR and coronary wire-derived FFR in patients with non-complex coronary artery disease (58, 93–95).

There are various software systems used to compute angio-derived fractional flow reserve (angio-FFR) (Table 1). The latest European Society of Cardiology (ESC) guidelines for managing chronic coronary syndrome have assigned a class I A recommendation to QFR computation as a viable alternative to FFR or iFR for evaluating the severity of epicardial artery stenosis during invasive coronary angiography (96). QFR is also recommended for assessing post-procedural outcomes following revascularization (96). Furthermore, in patients with chronic heart failure and a left ventricular ejection fraction (LVEF) greater than 35%, who are suspected of having CCS and exhibit a very high (>85%) pre-test likelihood of obstructive CAD, invasive coronary angiography with QFR is suggested as an alternative to FFR or iFR when deemed necessary (class IC) (96).

**Table 1:** Comparison of various commercially available angiography-derived FFR software.

Company	µFR	QFR	FFR angio	vFFR	caFFR	Angio-FFR	AutocathFFR
	Pulse medical	Medis/Pulse Medical	CathWorks	Pie Medical	Rainmed	Siemens	Medhub Ltd
Estimated	FFR	FFR	FFR	FFR	FFR	FFR	FFR
Required angio projections	1	2 projections >25° apart	2 projections >30° apart	2 projections	2 projections >30° apart	2 projections >30° apart	2 projections
Require pressure data	No	No	No	Needed	Needed	No	No
Side branches	Incorporated	Not incorporated	Incorporated	Not incorporated	Not incorporated	Incorporated	NA
Computation model	Kirkeeide	Lance Gould equation	Electric circuit model	Simplified Navier–Stokes	Simplified Navier–Stokes	AI based	AI based
Studies	Tu et al.	FAVOR pilot FAVOR II China FAVOR II EJ FAVOR III	FAST-FFR	FAST FAST II	FAST-FFR FLASH II	Omori et al.	Ben-Assa E et al. (188)
C-statistics for predicting FFR ≤ 0.8	0.97	0.92–0.96	0.94	0.93	0.98	0.90	0.93
Mean time to computation	67 s	4.36 min	2.7 min	NA	4.5 min	NA	45 s

AI, artificial intelligence; caFFR, coronary angiography-based fractional flow reserve; FAVOR, functional diagnostic accuracy of QFR in online assessment of coronary stenosis; FAST, fast assessment of stenosis severity; FFR, fractional flow reserve; µFR, Murray law-based fractional flow ratio; NA, not available; QFR, quantitative flow ratio; vFFR, vessel fractional flow reserve.

In Europe, there are currently eight angio-FFR software systems. These are discussed in the Supplementary Material.

### QFR as a surrogate for FFR to decide on revascularization in coronary stenoses

The American and European Societies of Cardiology define a significant lesion as one with more than 50% diameter stenosis in a major coronary artery (97, 98). However, a disagreement between anatomical and physiological assessments is observed in approximately 20% of lesions with QCA-estimated diameter stenosis over >70% and in half of the lesions with diameter stenosis between 50% and 70% when using FFR (89, 99). Although pressure wire assessment remains the gold standard for detecting ischemia-causing coronary lesions, several methods have been developed to estimate FFR from conventional angiography without the need for a pressure wire, demonstrating excellent correlation with wire-based measurements. QFR eliminates the need for intracoronary wires, making the procedure faster and safer for patients. Studies such as FAVOR I, FAVOR II China, and FAVOR II Europe-Japan have demonstrated a strong agreement between QFR and FFR in assessing coronary stenosis severity (93, 95, 100). In addition, numerous studies have shown excellent diagnostic performance for angio-FFR, with AUCs ranging from 0.93 to 0.97 (58, 100–103). A Bayesian bivariate meta-analysis of 13 studies, encompassing 1,842 vessels, evaluated the diagnostic accuracy of angiography-derived FFR systems against pressure wire-based FFR. The analysis revealed a pooled sensitivity of 89% and specificity of 90%. The summary area under the ROC curve was 0.84, indicating a strong overall diagnostic performance for detecting hemodynamically significant lesions (Supplementary Figure S2). (104)

In the *post hoc* analysis of the SYNTAX II trial, QFRs were analyzable in 71.0% of lesions (836 lesions). The diagnostic performance of QFR for predicting binary wire-based ischemia was substantial, achieving an area under the curve of 0.81 and accuracy of 73.8%, with a positive predictive value of 85.9%.

A recent systematic review and network meta-analysis encompassing (104) 15 trials with 16,333 participants and a mean weighted follow-up of 34 months found that QFR was linked to a reduced risk of MACE compared to CA [risk ratio (RR) 0.68], FFR (RR 0.73), and iFR (RR 0.63). QFR was ranked first for MACE prediction, with an 88.1% probability of being the best. In contrast, FFR (RR 0.93) and iFR (RR 1.07) likely did not significantly reduce MACE risk compared to CA. The findings suggest that decisions to perform a PCI based on QFR were associated with a lower risk of MACE compared to CA, FFR, and iFR in a population with both stable coronary disease and acute coronary syndrome. However, these hypothesis-generating results should be confirmed through large, randomized, head-to-head comparison trials.

### Anonymous comparison of various angiography-derived fractional flow reserve software with pressure-derived physiological assessment

**Figure 8:**
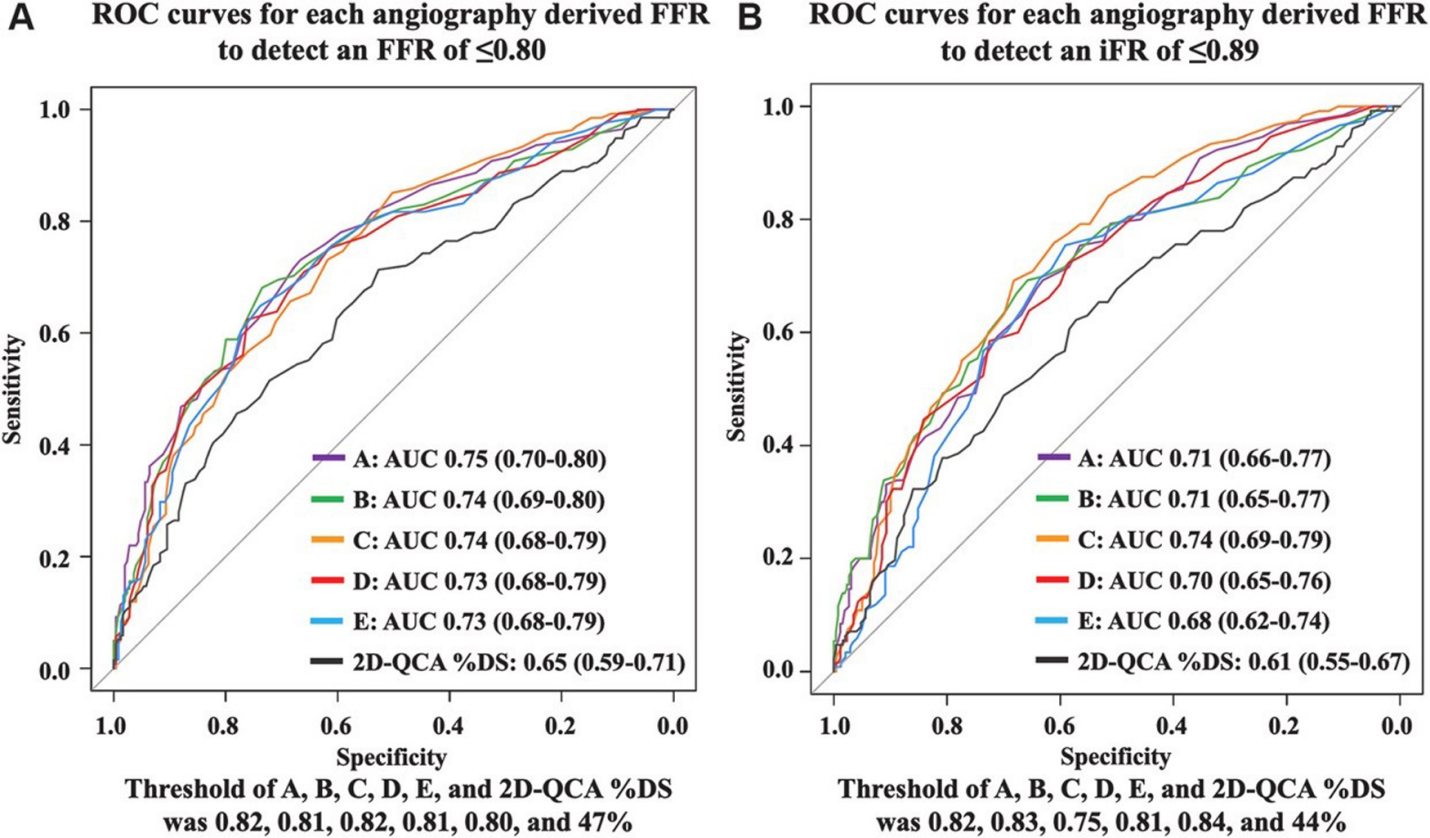
Anonymous comparison of various angiography-derived fractional flow reserve software with pressure-derived physiological assessment. ROC curves for each angiography-derived FFR against wire FFR and iFR. **(A)** ROC curves for each angiography-derived FFR to detect pressure wire-derived FFR ≤0.80. **(B)** ROC curves for each angiography-derived FFR to detect pressure wire-derived iFR ≤0.89 [Reproduced from Ninomiya et al. (105)].

This study focused on evaluating the diagnostic performance of five methods for deriving angio-FFR using four different software packages (QFR, vFFR, caFFR, and μFR with either one or two projections). An independent core laboratory conducted a head-to-head comparison of these assessment modalities within a prospective cohort, employing colocalized measurements of angio-FFR. PW-iFR and PW-FFR measurements were utilized as the reference standards. The study demonstrated that all five software methods achieved a high percentage of analyzable vessels: software A and B both at 100%, software C at 92.1%, software D at 99.5%, and software E at 92.1%. The AUC for predicting FFR ≤0.8 for each software was as follows: software A: 0.75, B: 0.74, C: 0.74, D: 0.73, and E: 0.73. In contrast, the AUC for two-dimensional QCA %DS was lower at 0.65. Each angio-FFR method significantly outperformed the two-dimensional QCA%DS in terms of AUC (58, 100). An independent core lab conducted a head-to-head comparison that demonstrated that the diagnostic accuracy of different angio-FFR software for predicting PW-FFR ≤0.80 was effective, offering better discrimination than two-dimensional QCA%DS. However, it did not achieve the diagnostic accuracy levels previously reported in validation studies from various vendors. Consequently, the inherent clinical value of “angiography-derived fractional flow reserve” needs validation in larger clinical trials (105) (Figure 8).

The ReVEAL iFR study, involving 400 patients with at least one stenotic coronary lesion, is currently examining the feasibility and accuracy of new physiological assessment software designed to predict wire-based FFR and iFR from a single angiographic projection, featuring highly automated and rapid measurement capabilities (106).

### Angiography-derived FFR stratified by left anterior descending and non-left anterior descending vessels

Although the FFR values and accuracy metrics could be vessel-dependent, in the FAST-FFR study, while lesion location (proximal, mid, or distal) did not have any impact (*p* = 0.71 and 0.12 for sensitivity and specificity, respectively), and the main vessel did not impact on the sensitivity (*p* = 0.99), it did have a slight effect on the specificity [98.7% for left anterior descending (LAD) artery, 86.3% for the left circumflex coronary artery (LCx), and 84.3% for right coronary artery (RCA); *p* = 0.046] for the FFR_angio_ (107). The FFR_angio_ value was similar to FFR_wire_ in the LAD territory (*p* = 0.37), whereas the FFR_angio_ value was lower in the LCx (*p* = 0.03) and RCA (*p* = 0.05) territories compared with FFR_wire_. When FFR_wire_ was separated into the cut-off zone (defined as 0.75–0.85) and beyond the cut-off zone (<0.75 or >0.85), both sensitivity and specificity tended to be numerically better beyond the cut-off zone (96.5% vs. 88.5%, *p* = 0.08 for sensitivity, and 93.3% vs. 85.1%, *p* = 0.10 for specificity) (107).

### Pre-procedural QFR for decision-making

Pre-procedural FFR is regarded as the reference standard for the evaluation of the physiological severity of obstructive CAD (108). Though angiography-based visual assessment remains the most widely used method to guide PCI, there has been emerging evidence that FFR-guided PCI reduces the number of stents implanted, with improved clinical outcomes as compared to angiography-guided PCI (109–111).

**Figure 9:**
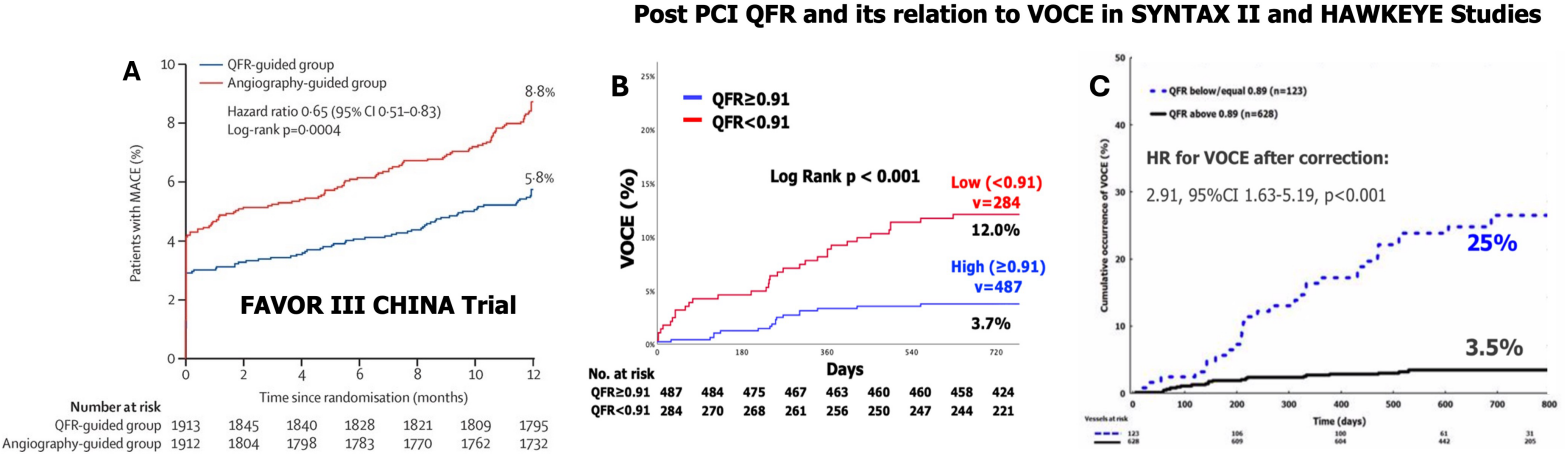
Results of the FAVOR III China multicenter randomized trial comparing angiographic quantitative flow ratio-guided vs. angiography-guided coronary interventions **(A)** and the clinical implications of quantitative flow ratio after a percutaneous coronary intervention in the SYNTAX II trial **(B)** and HAWKEYE study **(C)**. **(A)** Kaplan–Meier curves for the primary endpoints in the intention-to-treat population showed that among patients undergoing a PCI with a QFR-guided strategy for lesion selection had improved 1-year clinical outcomes compared with standard angiography guidance [Reproduced from Xu et al. (112)]; **(B)** Kaplan–Meier curves show the cumulative incidence of VOCEs (a composite of vessel-related cardiac death, vessel-related myocardial infarction, and target vessel revascularization) over 730 days of follow-up among the vessels with a low (<0.91) or high (≥0.91) post-PCI quantitative flow ratio (QFR) values in the SYNTAX II trial. **(C)** Black continuous line: vessels with post-PCI quantitative flow ratio (QFR) values ≤0.89. Blue dotted line: vessels with values >0.89. The cut-off of 0.89 was obtained by receiver-operating characteristic curve analysis for the best prediction of the VOCE [Reproduced from Kogame et al. (113)].

Cardiologists are currently increasingly using QFR to guide and plan PCI. A QFR value <0.80 suggests significant stenosis, requiring angioplasty. The FAVOR III CHINA was a multicenter, randomized control trial conducted at 26 centers in China. In this study, patients aged ≥18 years with stable or unstable angina or a myocardial infarction at least 72 h before screening were eligible if they had at least one coronary artery lesion with 50%–90% diameter stenosis and a reference vessel diameter of at least 2.5 mm by visual assessment (112). A total of 3,847 patients were randomly assigned to either a QFR-guided strategy (PCI only if QFR ≤0.80) or an angiography-guided strategy (112). After 1 year, the primary endpoint (death, myocardial infarction, or ischemia-driven revascularization) occurred in 5.8% of the QFR-guided group compared to 8.8% in the angiography-guided group (HR 0.65, 95% CI 0.51–0.83; *p* = 0.0004), largely due to fewer myocardial infarctions and ischemia-driven revascularizations in the QFR-guided group. The study demonstrated that QFR-guided PCI led to improved 1-year clinical outcomes compared to angiography guidance (112) (Figure 9A).

The AQVA trial was a multicenter RCT involving 300 patients (356 vessels) comparing QFR-based PCI with angiography-based PCI. The primary outcome was the rate of vessels with a suboptimal post-PCI QFR (≤0.90). QFR-based PCI was significantly better, showing an absolute difference of 8.5% and a relative reduction of 57% (*p* = 0.009) (114). The follow-up AQVA II trial (115) further demonstrated that in patients undergoing complex high-risk indicated procedures (CHIPs), procedural planning and guidance based on physiology (using either angiography or FFR) were superior to conventional angiography for achieving optimal post-PCI FFR values.

The Multivessel TALENT trial is a prospective, multicenter, randomized control trial comparing clinical outcomes between the ultra-thin Supraflex Cruz and SYNERGY drug-eluting stents in 1,550 patients with *de novo* 3VD without left main disease. Patients will be treated using a state-of-the-art PCI after selection based on their SYNTAX score II and heart team (HT) discussion; physiological evaluation of a stenotic lesion will be performed using the QFR and IVUS/OCT optimization post the PCI. All the patients will receive optimal medical therapy and will be followed up for 2 years after the index procedure. The primary endpoint is a patient-oriented composite endpoint (POCE) and includes all-cause death, any stroke, and any MI (116).

The PIONEER IV trial is an ongoing multicenter randomized controlled trial which will recruit 2,540 patients, from 30 European sites, in a 1:1 ratio to PCI guided by angiography-derived physiology or usual care, with unrestricted use in both arms of the healing-targeted Supreme sirolimus-eluting stent and 1 month of dual-antiplatelet therapy (DAPT) followed by 11 months of ticagrelor monotherapy. The primary outcome of the study is a POCE (composite of all-cause death, any stroke, any myocardial infarction, or any clinically and physiologically driven revascularization) with a non-inferiority risk difference margin of 3.2% at 1 year (117).

### QFR to guide PCI vs. CABG decisions in multivessel CAD

The DECISION QFR trial evaluated the feasibility of using QFR in HT discussions to determine the best revascularization strategy for patients with multivessel CAD (118). The study aimed to assess the agreement between two separate HTs on treatment planning based on QFR or FFR estimations. The primary endpoint was consensus on a revascularization strategy between the HTs. QFR/FFR values were used to derive the functional SYNTAX score (FSS), which subtracted non-flow-limiting stenoses (QFR/FFR > 0.80) from the angiography-based aSS. This non-invasive FSS was then incorporated into the SYNTAX score II 2020, which combines coronary anatomy, physiological impact, and patient clinical factors. The trial involved 248 multivessel CAD patients, and Cohen's kappa for agreement on revascularization strategies between QFR and FFR approaches was 0.73 (95% CI: 0.62–0.83). There was substantial agreement in identifying target vessels for PCI and CABG (Cohen's kappa = 0.72 for both). Even after reviewing the FFR data, the QFR-based team maintained consistent revascularization recommendations (Cohen's kappa = 0.95) (118). The study concluded that QFR effectively guided HT discussions and substantially aligned with FFR in treatment planning for multivessel CAD, marking one of the first assessments of vessel-level QFR for HT decision-making (118).

### QFR for the revascularization of non-culprit vessels

This is a topic of ongoing discussion. The Functional Assessment in Elderly MI Patients With Multivessel Disease (FIRE) trial (NCT03772743) randomized 1,445 older MI patients to either culprit-only or physiology-guided complete revascularizations. In a pre-specified subanalysis, QFR was measured for 903 non-culprit vessels from 685 patients in the culprit-only arm. Overall, 366 (40.5%) non-culprit vessels had a QFR ≤0.80, with a significantly higher incidence of vessel-oriented clinical events (VOCEs) (22.1% vs. 7.1%; *p* < 0.001) at the 1-year follow-up. QFR ≤0.80 was an independent predictor of VOCEs (HR: 2.79; 95% CI: 1.64–4.75).

### Post-procedural QFR predicts clinical outcomes

Post-PCI FFR is recognized as an independent predictor of long-term clinical outcomes (119) with an optimal cut-off value ranging from 0.86 to 0.96 correlating with various clinical events (111, 120–122).

In a *post hoc* analysis of the SYNTAX II study, which focused on patients with *de novo* 3VD treated with advanced PCI, post-PCI QFR was identified as the strongest independent predictor of VOCEs at 2 years (113). Out of 968 vessels treated with PCI, post-PCI QFR was analyzable in 771 (79.6%), and 52 (6.7%) VOCEs occurred. The average post-PCI QFR value was 0.91 ± 0.07. The diagnostic performance of post-PCI QFR in predicting 2-year VOCEs was moderate, with an AUC of 0.702. The optimal post-PCI QFR cut-off for predicting 2-year VOCE was 0.91, with a sensitivity of 65.2% and specificity of 63.5%. The 2-year incidence of VOCEs was significantly higher in vessels with post-PCI QFR <0.91 (*n* = 284) compared to those with post-PCI QFR ≥0.91 (*n* = 487), with rates of 12.0% vs. 3.7%, respectively. This corresponds to a hazard ratio of 3.37 (95% confidence interval: 1.91–5.97; *p* < 0.001), indicating a substantially higher risk of VOCEs in vessels with a post-PCI QFR below 0.91 (Figure 9B).

A recent vessel-based analysis from the first 775 patients in the Multivessel TALENT trial revealed that 76% of cases had favorable post-PCI QFR values (QFR ≥0.91). Applying the logistic regression model from the SYNTAX II trial, the predicted rate of VOCEs based on post-PCI QFR was 6.1%, with prediction intervals ranging from 4.8% to 7.4% (123) (Supplementary Figure S3).

The HAWKEYE study, an international, multicenter trial, assessed 751 vessels in 602 patients who underwent complete revascularization with a successful PCI and stent implantation. QFR was measured at the conclusion of the procedure. Vessels that experienced a vessel-oriented clinical event during follow-up had significantly lower post-PCI QFR values compared to those without an event [median 0.88 (IQR: 0.81–0.99) vs. 0.97 (IQR: 0.93–0.99); *p* < 0.001]. A post-PCI QFR ≤0.89 was linked to a threefold increased risk of vessel-related events (HR: 2.91; 95% CI: 1.63–5.19; *p* < 0.001) (111). Several ongoing studies are further investigating QFR's clinical value (Table 2). A few of the important ones are summarized below (Figure 9C).

**Table 2:** Ongoing studies using angiography-derived FFR.

Investigation topic	Type of trial	Patient no. and country
FAVOR III Europe-Japan trial QFR vs. FFR in patients with CCS + intermediate stenosis and ACS + intermediate stenosis in non-culprit vessel	Multicenter RCT	2,000 patients NCT03729739
PIONEER IV trial QFR guidance vs. usual care guidance in all-comer patients referred to angiography with at least one significant lesion (DS ≥ 50%) for PCI	Multicenter RCT	2,540 patients Europe NCT04923191
AQVA trial QFR-based virtual PCI vs. CAG-guided PCI	Two centers, RCT	300 patients Italy NCT04664140
Multivessel TALENT trial QFR-guided revascularization in multivessel CAD	Multicenter RCT of Supraflex vs Synergy in multivessel CAD	1,550 Patients Europe NCT04390672
FAST III trial vFFR-guided vs. FFR-guided coronary revascularization in intermediate coronary artery lesions	Multicenter RCT	220 patients China NCT04931771
QFR-guided revascularization of non-culprit vessels in STEMI patients with multivessel CAD QFR (Pulse)-guided PCI vs. CAG-guided PCI	Multicenter RCT	1,016 patients China NCT04259853
FAVOR V AMI μFR + RWS-guided revascularization vs. CAG-guided PCI of non-culprit vessels in STEMI patients with multivessel CAD	Multicenter RCT	5,000 patients NCT05669222
	Multicenter RCT	792 patients China NCT03977129
UNIQUE-DCB-I study Safety and efficacy of DCB therapy for *de novo* lesions under the guidance of QFR in CAD patients	Multicenter RCT	220 patients China NCT04104854
UNIQUE-DCB-II study Safety and efficacy of DCB therapy for ISR under the guidance of QFR	Multicenter RCT	220 patients China NCT04119986
QFR-based-CABG vs. angio-guided CABG	Single-center, superiority RCT	208 patients China NCT03770520

ACS, acute coronary syndrome; CABG, coronary artery bypass grafting; CAD, coronary artery disease; CAG, coronary angiography; CCS, chronic coronary syndrome; DCB, drug-coated balloon; DS, diameter stenosis; FAVOR, functional diagnostic accuracy of QFR in online assessment of coronary stenosis; FAST, fast assessment of stenosis severity; FFR, fractional flow reserve; ISR, in-stent restenosis; µFR, Murray law-based fractional flow ratio; NA, not available; PCI, percutaneous coronary intervention; QFR, quantitative flow ratio; RCT, randomized control trial; RWS, radial wall strain; STEMI, ST segment elevation myocardial infarction; vFFR, vessel fractional flow reserve.

### Pullback pressure gradient

The ultimate goal of PCI is to improve clinical outcomes. The results obtained post-PCI depend on the disease patterns according to pathophysiological phenotypes that can be classified as predominantly diffuse or focal disease. The pullback pressure gradient (PPG) was introduced to differentiate these two patterns by Collet et al., using a motorized coronary pressure pullback device that withdraws an FFR wire during continuous hyperemia (124). The PPG index is calculated by determining three key parameters: the maximum pressure gradient within a 20 mm segment of the artery, the total pressure gradient across the entire lesion, and the length of the lesion itself (124). A PPG value closer to 1.0 is suggestive of focal disease and a value close to 0 is suggestive of diffuse disease. A PPG derived from invasive manual FFR pullback has been shown to identify patients who would benefit the most from a PCI in terms of angina relief (125). Moreover, PPG Global, a large-scale prospective study, showed that the PPG also correlates with the safety of a PCI as patients with a low PPG (diffuse disease) had double the rate of peri-procedural myocardial infarction than patients with a high PPG (126).

**Figure 10:**
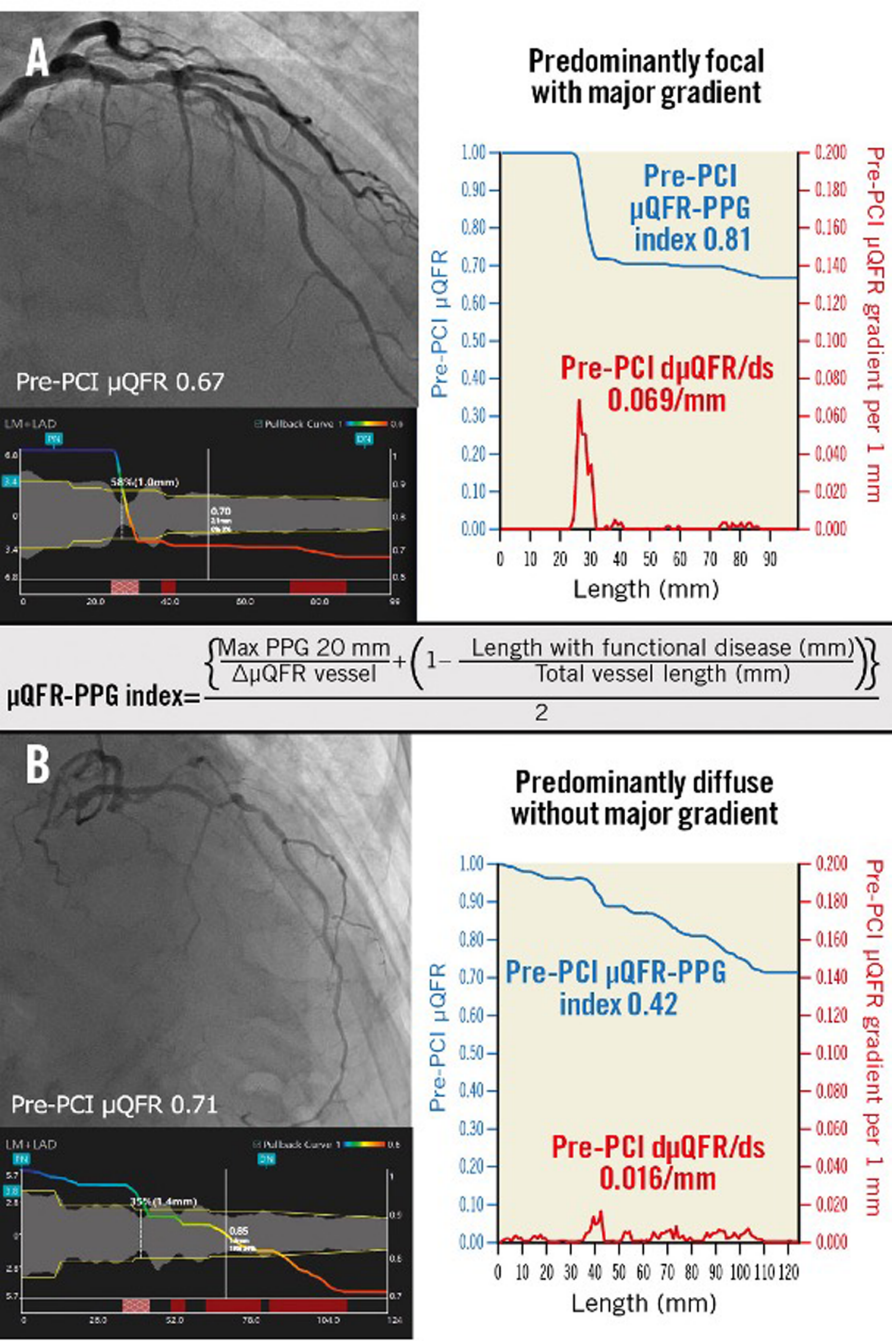
Pathophysiological CAD patterns assessed by PPG acquired from the pre-PCI angiogram. Red curves in the graphs show pressure drop per 1 mm (dµFR/ds). Cumulative pressure drop is represented as blue curves. **(A)** The vessel shown has predominantly focal (µFR-PPG ≥0.78) disease with a major gradient (dµFR/ds ≥0.025/mm). **(B)** The vessel shown has predominantly diffuse disease without a major gradient. In this case, the PPG acquired from the pre-PCI angiogram was 0.42 (<0.78), with the widespread distribution of pressure drop along the target vessel. CAD, coronary artery disease; dµFR/ds, instantaneous µFR ratio gradient per unit length; PCI, percutaneous coronary intervention; PPG, pullback pressure gradient; µFR, Murray law-based quantitative flow ratio [Reproduced from Kotoku et al. (128)].

The PPG can be estimated using a FFR wire or angiography-derived FFR software. It was recently shown by Kotoku et al., that pathophysiological patterns could be characterized by µFR virtual pullback without a pressure wire pullback (128) (Figure 10).

### Applications of the PPG

The PPG index has been used to guide a PCI and to predict post-PCI FFR. This is clinically relevant since the change of FFR after a PCI correlates directly with angina relief and the absolute post-PCI FFR is associated with worse clinical outcomes. It can also predict target lesion failure and patients’ symptoms and quality of life post-PCI (129). It has also been hypothesized that it can predict plaque morphology. A recent study has shown that the focal disease type is associated with an increased plaque burden and vulnerable lipid-rich morphology whereas the diffuse disease type exhibits a more stable phenotype and increased calcium burden (130). This, however, was not confirmed by the study by Kotoku et al. which showed no association between plaque morphology and pathophysiology derived from the PPG (128). The authors measured coronary physiology patterns in 206 patients, recruited from the ASET-JAPAN study. Plaque composition was assessed in stented and non-stented segments using IVUS or OCT imaging pre- and post-PCI with the μQFR-PPG. Patients with the diffuse disease had a larger plaque burden and smaller lumen area in non-stented segments compared to those in the focal disease group but there was no difference in the plaque composition.

The rate of target vessel failure after a PCI was significantly higher in patients with diffuse disease (QFR-based PPG <0.78) compared with those with focal disease (QFR-based PPG ≥0.78) (127, 131). The first studies examining the clinical relevance of this index are promising; however, further research is needed to clarify whether this index is a surrogate of the plaque burden or a meaningful marker of coronary pathology that carries independent prognostic implications (124, 127).

## Co-registration of physiology with angiography

**Figure 11:**
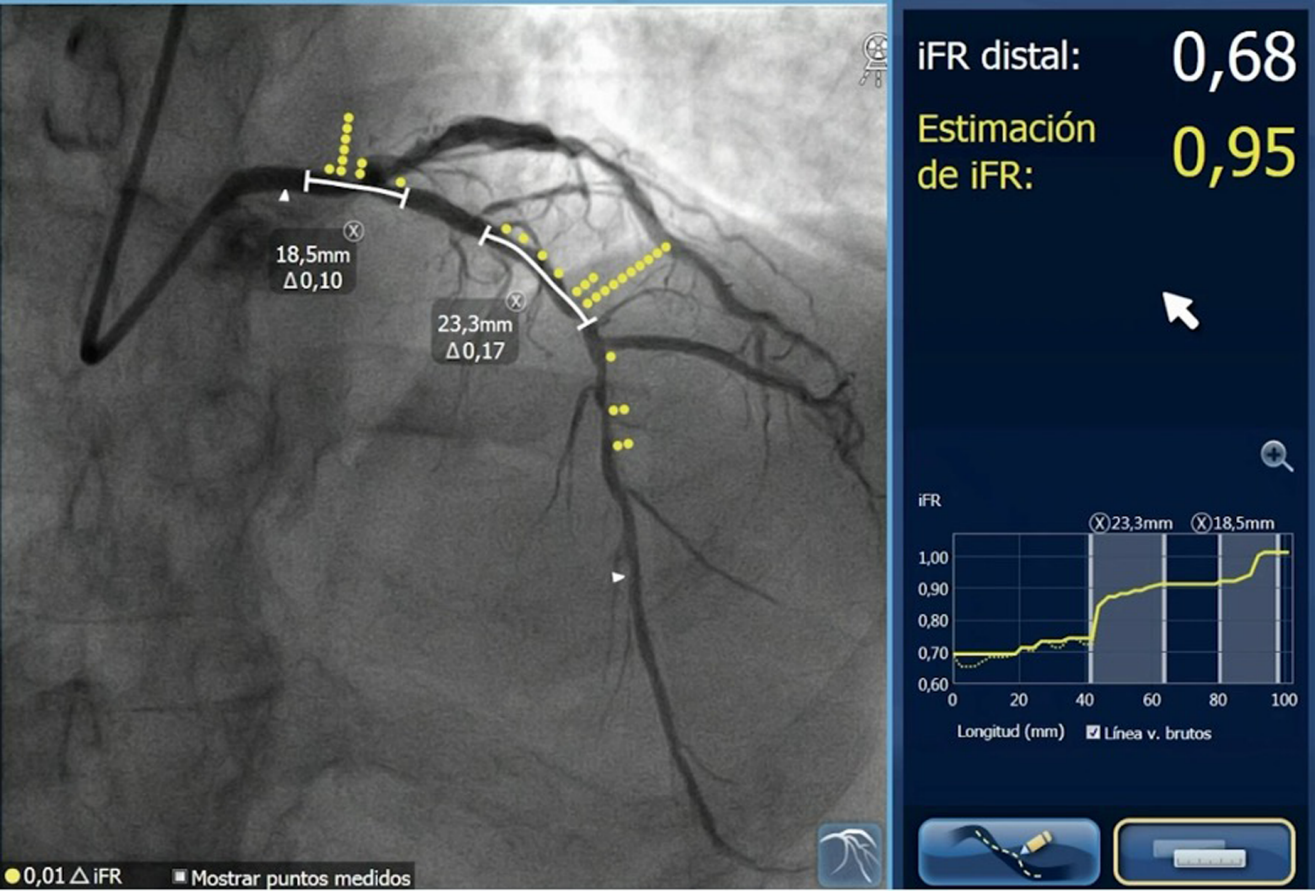
Longitudinal iFR mapping co-registered with coronary angiography showing flow-limiting disease in the left main stem and left anterior descending artery with a physiologically significant iFR of 0.68, and predicted post-PCI iFR of 0.95 after successful treatment of the selected segment.

Integrating longitudinal vessel physiology with coronary angiograms enables the precise localization of flow-limiting atherosclerotic disease, improving procedural planning (132). The co-registered map highlights regions of pressure loss, guiding the optimal placement of the stent during a PCI and accurate lesion length measurements (133). This technology, which can be used with iFR, is especially helpful in strategizing interventions for tandem lesions and assessing diffuse disease. For the same PPG value, some vessel hemodynamics may still not respond to a PCI, indicating that additional clinical judgment is needed (Figure 11).

## Index of microvascular resistance: invasive assessment of coronary microcirculation

### Coronary microvascular dysfunction

Coronary microvascular dysfunction is responsible for angina symptoms in 30%–40% of patients and is described with the term non-obstructive coronary artery disease (NOCA) (134–136). This has given birth to the term ischemia in non-obstructive coronary artery disease (INOCA). Several conditions lead to coronary microcirculatory dysfunction, which impacts both the structure and function of the coronary microcirculation. Structural changes involve arteriolar and capillary narrowing, perivascular fibrosis, and a reduction in capillary density (137). Functional causes of coronary microvascular dysfunction are attributed to an increased baseline flow leading to an imbalance between resting and vasodilatory response. The Doppler wire, introduced in 1987, and the pressure wire in 1991, enabled invasive coronary physiology assessments (138). In 1993, the Thorax Center group in Rotterdam first demonstrated the feasibility of *in vivo* intracoronary measurements using guidewires with combined pressure–velocity sensors. Pressure–volume loops during hyperemia also validated the functional relationship between epicardial stenosis and microcirculation (139). Today, two invasive methods are available for this evaluation: Doppler-based tools and thermodilution-based tools, both bolus and continuous. An index of microvascular resistance (IMR) value of less than 25 indicates healthy microvascular circulation, regardless of epicardial disease. In ST segment elevation myocardial infarction (STEMI) patients, an IMR >40 following a primary PCI predicts all-cause mortality, readmission for heart failure, and MACE (140, 141).

### Vogel technique

Vogel developed a method for enhancing images in selective coronary angiography and assessing coronary flow reserve. This technique was validated in dogs by comparing digital flow ratio estimates with electromagnetic flow (EMF) ratio measurements, demonstrating accurate results and high reproducibility (142). This technique visually represents the spatial distribution and timing of the contrast medium during its arterial, myocardial, and venous phases through functional images that utilize simultaneous modulation of color and intensity. The myocardial contrast appearance time obtained from this method is inversely related to regional blood flow. It provides improved temporal separation of the different phases of contrast medium transit, with the data presented in color- and intensity-modulated functional images. The relative changes in regional myocardial contrast appearance time, measured by this technique, were found to correlate with coronary blood flow assessed via the thermodilution method in the coronary sinus and great cardiac vein (143). This approach offers several advantages, including precise visualization of the coronary artery tree and ventriculography, along with reduced contrast volume and radiation exposure. Suryapranata et al. demonstrated that a reactive hyperemic response and coronary flow reserve—calculated by digitally subtracting contrast medium appearance time and density—were independent predictors of regional myocardial function recovery at a median follow-up of 10 days in patients with acute MI treated with a PCI (144).

### Angiography-derived IMR

Two recent proof-of-concept studies investigated angiography-derived indices of microcirculatory resistance. De Maria et al. developed and validated an angiography-derived microcirculatory resistance index (IMR_angio_) in 45 STEMI patients undergoing a primary PCI, showing a strong correlation (*ρ*: 0.85, *p* < 0.001) between conventional IMR and IMR_angio_ measured just before stenting during the procedure (145).

Tebaldi et al. validated another angiography-based index of microcirculatory resistance (A-IMR), using conventional IMR as the reference standard in patients with chronic coronary syndrome and intermediate lesions in the LAD artery, demonstrating a strong correlation between the two indices (146). Meanwhile, Mejia-Renteria et al. developed an adenosine- and wire-free IMR method (angio-IMR) that was tested in 115 vessels from 104 patients, which also showed a good correlation (*r* = 0.70, *p* < 0.001) with invasive IMR (147).

Finally, Choi et al. confirmed the prognostic significance of elevated angio-IMR, calculated through computational flow and pressure simulations, in two cohorts of STEMI patients followed for 10 years. Their findings indicated that patients with an angio-IMR >40 faced a significantly higher risk of cardiac death and hospitalization for heart failure (148).

A recent study explored the relationships between corrected TIMI frame count (cTFC) and invasive coronary functional testing (CFT) for coronary microvascular dysfunction in 508 adults with non-obstructive CAD. Patients exhibiting slow flow were more likely to show abnormal IMR (36% vs. 26%; *p* = 0.019) but were less likely to present with abnormal CFR (28% vs. 42%; *p* = 0.001), with no significant difference in coronary microvascular dysfunction (CMD) (46% vs. 51%). The cTFC demonstrated weak correlations with baseline coronary blood flow (*r* = −0.35), CFR (*r* = 0.20), and IMR (*r* = 0.16) (149). In multivariable models, slow flow was linked to reduced odds of abnormal CFR (adjusted OR: 0.53) (149).

## Coronary angiography-derived wall shear stress

Flow patterns and the distribution of the wall shear stress (WSS) in particular appear to influence endothelial function and determine atherosclerotic disease progression (150, 151). Traditionally the WSS computation relies on the accurate vessel reconstruction derived from the combination of intravascular imaging and angiographic data which are fused to generate 3D models that are then processed using computational fluid dynamics techniques. However, the entire process is laborious, time-consuming, and requires expertise (152–155). To overcome this limitation, 3D-QCA has been proposed for WSS computation with the first studies providing promising results. In one of the first studies, Wellnhofer et al. examined the impact of two lumen reconstruction methods—one assuming circular cross-sections and the other elliptical—using varying resolutions for cross-section reconstructions along the vessel axis (156). The study utilized three right coronary arteries: one normal, one with “obstructive” atherosclerosis, and one with “dilated” atherosclerosis. Vessel volume reconstruction was carried out using 3D data from a validated 3D angiographic model of vessel cross-sections and axes. The difference in vessel volumes calculated by the two methods was under 1%, while the calculated pressure loss varied between 2.5% and 8.5%. In addition, the distributions of the WSS histograms were nearly identical and exhibited strong cross-correlation (0.91–0.95) (156).

Goubergrits et al. demonstrated that biplane angiography-based reconstructions can be utilized for WSS profiling of coronary arteries (157). In their study, a silicon model of the left coronary artery (LCA) was obtained through biplane angiography. The geometry was reconstructed using commercial CAAS 5.2 QCA 3D software and compared to an original model, which was an optically digitized postmortem vessel cast. Steady flow simulations were conducted using the commercial CFD program FLUENT. The comparison of calculated WSS indicated a strong correlation in the histograms (*r* = 0.97) and good agreement across four modalities, with mean WSS values of 0.65 Pa in the original model, 0.68 Pa in the CT-based model, 0.67 Pa in the MRI-based model, and 0.69 Pa in the biplane angiography-based model (157).

Timmins et al. compared biplane angiographic and IVUS-derived reconstructed coronary geometries to assess their agreement regarding geometry, computed WSS, and the association between WSS and CAD progression (158). They collected baseline and 6-month follow-up angiographic and IVUS imaging data from patients with non-obstructive CAD (*n* = 5). The study found strong agreement between the angiographic and IVUS-derived coronary geometries, with computed absolute time-averaged WSS (TAWSS_ABS_) values significantly higher in the IVUS-derived geometries. However, evaluations of relative TAWSS (TAWSS_REL_) showed improved agreement within defined zones of equivalence. Associations between virtual histology (VH)-IVUS defined CAD progression and WSS from either angiographic or IVUS-derived data showed poor agreement when examining TAWSS_ABS_, but better concordance when assessing the association with TAWSS_REL_ data (158).

In a retrospective study of 548 patients with borderline negative FFR (FFR: 0.81–0.85), 3D-QCA-derived WSS was assessed in 293 lesions (286 patients) with appropriate angiographic views. Over a median follow-up period of 49.4 months, 37 events were documented. The culprit lesions exhibited greater area stenosis (AS) (66.1% vs. 54.8%, *p* < 0.001), smaller MLA (1.66 vs. 2.10 mm^2^, *p* = 0.011), and higher maximum WSS (9.0 vs. 5.0 Pa, *p* < 0.001) compared to those that remained stable. Multivariable analysis identified AS (HR: 1.06, *p* = 0.001) and maximum WSS (HR: 1.08, *p* = 0.012) as the only independent predictors of the primary endpoint. Lesions with increased AS (≥58.6%) under high WSS (≥7.69 Pa) had a higher progression and event rate (27.8%) compared to those with low AS under high WSS (7.4%) or those exposed to low WSS with either increased (12.8%) or low AS (2.7%, *p* < 0.001) (49). Another study highlighted the predictive ability of a new WSS-derived parameter, topological shear variation index (TSVI), which demonstrated that 3D-QCA-based WSS analysis is feasible for identifying lesions likely to be culprits for future MIs. This combination of area stenosis, pressure gradients, and WSS effectively predicted MI occurrence, with TSVI showing strong predictive power for detecting lesions at risk of rupture and MI (159).

Similar were the findings of a report that combined 3D-QCA-derived WSS and plaque morphology derived by VH-IVUS to predict vulnerable plaques (160). This study evaluated baseline VH-IVUS and angiographic data from 28 lipid-rich lesions that resulted in major adverse cardiovascular events or necessitated revascularization (MACE-R) during a 5-year follow-up, alongside 119 lipid-rich lesions from a control group that remained stable. The segments analyzed by VH-IVUS at baseline were reconstructed using 3D-QCA software. Blood flow simulations were conducted on the obtained geometries, estimating the pressure gradient across the lipid-rich plaque and the mean endothelial shear stress (ESS) values in 3-mm segments (160). MACE-R lesions were longer, had smaller MLA, increased PB, were exposed to higher ESS, and exhibited a higher pressure gradient. In multivariable analysis, PB (hazard ratio: 1.08; *p* = 0.004) and the maximum 3-mm ESS value (hazard ratio: 1.11; *p* = 0.001) were independent predictors of MACE-R. Lesions exposed to high ESS (>4.95 Pa) with a high-risk anatomy (MLA < 4 mm^2^ and PB > 70%) had a higher MACE-R rate (53.8%) than those with a low-risk anatomy exposed to high ESS (31.6%) or those exposed to low ESS who had high- (20.0%) or low-risk anatomy (7.1%; *p* < 0.001) (160). The promising results of the above analysis motivated the industry to develop dedicated software for 3D-QCA-based WSS computation. The CAAS Workstation WSS prototype software (Pie Medical Imaging, Maastricht, the Netherlands) introduced for this purpose allows the measurement of the WSS values seamlessly within only a few minutes.

A recent study by Tufaro et al. (161) demonstrated strong agreement between the WSS estimations provided by the CAAS workstation software and those calculated using conventional CFD analysis in 3D-QCA reconstructions. In addition, a study by Kageyama et al. evaluated inter-core lab reproducibility and found excellent agreement between the estimations from the two core laboratories (162).

## Coronary angiography-derived radial wall strain

Recently, a coronary angiography-derived radial wall strain (RWS) measurement method was introduced to quantify vessel deformation and indirectly assess the lesion-specific biomechanical features (163). Studies have shown that the RWS is associated with plaque vulnerability and predicts disease progression and clinical outcomes in non-flow-limiting lesions. In a recent study involving 484 vessels from 351 patients with coronary artery disease, increased maximum relative wall shear stress (RWS_max_) was linked to a higher risk of FFR ≤0.80 and high-risk plaques (HRP) on CCTA, even after adjusting for clinical and angiographic characteristics (all *p* < 0.05) (163). High RWS_max_ was also associated with an elevated risk of TVF (HR: 1.23, *p* = 0.022), with an optimal cut-off of 14.25%. RWS_max_ >14% emerged as a predictor of TVF after adjusting for FFR or HRP components on CCTA (all *p* < 0.05). Furthermore, when high RWSmax was included alongside FFR ≤0.80 or HRP, a trend of increasing outcomes was observed (all *p* for trend <0.001) (163).

## Angiography-based four-dimensional superficial wall strain and stress

A novel method for four-dimensional superficial wall strain and stress (4D-SWS) has been developed from arterial motion observed in coronary angiography. Unlike conventional finite element analysis, which uses estimated pulsatile pressure, the 4D-SWS approach calculates the dynamic mechanical state of the superficial wall *in vivo*, directly linking it to plaque rupture or stent fractures (164). Validation with *in silico* models showed that the distribution and maximum values of superficial wall stress closely matched those obtained through traditional finite element analysis. *In vivo* deformation was validated in 16 coronary arteries by comparing centerlines predicted by the 4D-SWS method to actual centerlines reconstructed from angiograms at a random time point, demonstrating strong agreement in morphology (scaling: 0.995 ± 0.018; dissimilarity: 0.007 ± 0.014). *In silico* models with softer plaques and larger plaque burdens exhibited greater variations in mean lumen diameter and higher superficial wall stress. In over half of the patients (*n* = 16), the maximum superficial wall stress was found at the proximal lesion shoulder. Furthermore, in three patients who later experienced acute coronary syndrome, the sites of plaque rupture coincided with areas of highest superficial wall stress on baseline angiography. Ongoing studies aim to pinpoint vulnerabilities in coronary bypass grafts and explore the biomechanical mechanisms of arterial remodeling and aneurysm formation. Future advancements will integrate rapid computational techniques for real-time assessment of superficial wall strain and stress in catheterization laboratories (164).

## QFR as part of Academic Research Consortium II to adjudicate clinically indicated target vessel revascularization

In clinical trials, events should be adjudicated independently and blindly by a clinical events committee (CEC) (165). There have been a variety of methods available for CEC adjudication, however, despite this, biases and inconsistencies remain a concern in conventional CEC adjudication (166). In cardiovascular trials, clinically indicated TLR (CI-TLR) is commonly used as a composite endpoint. Thus CI-TLR needs to be accurately adjudicated, as it significantly influences the clinical outcomes of trials. In most studies, the adjudication of TLR is based on a visual estimation of stenosis severity or QCA. Currently, a widely accepted criterion for CI revascularization is a 50%–70% stenosis of the target lesion or target vessel, as assessed by QCA by an independent core lab (167). In the evaluation of lesion severity, QFR is superior to QCA and has been utilized for CEC adjudication. The study of Wang et al. was the first to evaluate the feasibility of adjudicating events using QFR and compare it with CEC-based adjudication. This study showed that there was a fair agreement between CEC and QFR-based adjudication and that CEC adjudication appears to overestimate CI revascularization as compared to QFR adjudication. The Academic Research Consortium II document on clinical end points in coronary intervention trials recommends physiological evaluation using the iFR, FFR, or QFR for the adjudication of CI revascularization (167).

## Coronary CT angiography

CT coronary angiography has emerged as the primary non-invasive method for assessing coronary obstructive disease. This modality offers a thorough assessment of atherosclerotic plaques, encompassing both calcific and non-calcific types (168, 169). It also has a high diagnostic performance for coronary stenosis assessment when compared with QCA (170, 171), transitioning from qualitative to quantitative analysis. Beyond luminal stenosis assessment, however, CT coronary angiography provides information on plaque composition that is inferior to intravascular imaging. Photon counting CT (PCCT) was introduced to overcome the limitations of conventional CT in plaque analysis and enhance its role in vulnerable plaque detection. Particularly beneficial for coronary bifurcation lesions, PCCT enables vessel reconstruction, allowing evaluation of the spatial relationship between the plaque and the side branch and has the potential to predict vessel wall response and side branch occlusion.

When utilizing CT coronary angiography, the anatomical site of atherosclerotic plaque can be correlated with the risk associated with a bifurcation PCI, especially for non-calcified plaque (172). In cases of severe calcification, CT coronary angiography helps stratify calcified plaque based on arc, length, and thickness, aiding in predicting PCI risks and recommending upfront use of calcium plaque modification techniques such as lithotripsy (173).

In addition, CCTA quantifies the myocardial mass subtended by the side branch, offering insights into its clinical significance (174). Coronary blood flow simulation using fractional flow reserve (FFR_CT_), enables assessment of coronary ischemia, which is crucial for assessing bifurcation lesions and selecting patients for PCI. FFR_CT_ facilitates the selection of patients for PCI and has value in PCI planning as it allows measurement of the trans-lesional pressure gradients across the various segments including bifurcation lesions (128). In this way, FFR_CT_ can be used to facilitate virtual stenting and predicting physiological outcomes in both main vessel and side branches (175).

Current FFR_CT_ planner technology can allow provisional and two-stent strategies, thus enabling comprehensive morphological and physiological planning for PCI (176, 177). This brings CCTA to the frontline of interventional cardiology as it allows not only diagnosis but also pre-procedural planning and intra-procedural guidance. The study by Collet et al. (175) demonstrated that in patients with left main or three-vessel disease, heart team decision-making based on coronary CTA showed high agreement with the decision derived from conventional coronary angiography, suggesting the potential feasibility of treatment decision-making and planning based solely on non-invasive imaging and clinical information (Cohen's Kappa 0.82, 95% confidence interval 0.74–0.91).

It stands out in its application in bifurcation lesions, wherein it non-invasively provides relevant information about the best projections, prognostic index, plaque extension and composition, and mass subtended by the side branch.

### Updated coronary vessel sizing by angiography

Coronary angiography is inherently two-dimensional and thus has limitations in the assessment of bifurcation lesions. The most important aspect is identifying and acquiring the optimal angulation to visualize critical information such as the distribution of the plaque burden. A single projection view may be deficient in providing all the relevant information, for instance, a certain view may be best for evaluating lesion length, whereas another provides insights into the side branch ostium. Coronary CTA emerges as an alternative option, especially in bifurcation lesions. The rotational capabilities of three-dimensional CT provide precise angulations, improving the visualization of lesions. CCTA before a PCI may identify the most favorable fluoroscopic view that will optimize exposure of the 3D bifurcation structure on a 2D angiographic projection during the procedure (178).

This addresses the primary limitation of coronary angiography for visualizing bifurcation lesions and it also cuts down on the contrast volume and procedural time.

### Coronary computed tomography-derived fractional flow reserve

Coronary computed tomography-derived fractional flow reserve (FFRCT; HeartFlow FFRCT Analysis) utilizes CFD to solve Navier–Stokes equations, simulating flow, pressure, and velocity during rest and hyperemia. Unlike fractional coronary angiography (FCA) which relies on invasive angiograms, FFRCT uses a supercomputer for full CFD analysis. Coronary 3D modeling is obtained through conventional CCTA, with patient-specific boundary conditions determined by lumped models for the heart (inlet) and coronary microcirculation (outlet). To mitigate supercomputer dependence, reduced-order and steady-state models average the Navier–Stokes equations over vessel cross-sections. In addition, machine learning models using artificial intelligence algorithms have emerged to calculate stenosis severity via a multilayer neural network architecture and offline training (179). Several studies have validated FFRCT using wire-based FFR as the gold standard reference. The DISCOVER-FLOW study found a good correlation between FFRCT and FFR, with FFRCT outperforming CCTA alone (180). The DeFACTO study reported similar results (181). The HTNXT study, which included high-quality CCTA (with beta-blocker and nitroglycerine administration) before ICA, used a refined FFRCT algorithm that significantly enhanced the diagnostic accuracy and specificity compared to CCTA alone (182). A study by Omori et al. indicated that the higher AUC for FFRCT values measured 1–2 cm distal to the stenosis was driven by findings in the LAD artery, rather than the LCx or RCA. This is likely due to the larger myocardial territory supplied by the LAD artery, resulting in greater flow and pressure gradients between segments 1–2 cm distal to the stenosis and more distant segments compared to non-LAD lesions. Further research is needed to determine the optimal FFRCT measurement location for LAD vs. non-LAD lesions (183).

The PLATFORM study randomized 584 patients with planned ICA to receive either usual care or CCTA/FFRCT. Results showed that 61% of ICA procedures were deferred after CCTA/FFRCT, with low clinical event rates at 90 days in both groups (184). One-year outcomes indicated that CCTA/FFRCT-guided care was associated with lower costs and equivalent quality of life and clinical outcomes (185).

The ADVANCE registry enrolled 5,083 patients with coronary atherosclerosis identified on CCTA to evaluate the clinical significance of functionally significant stenosis using FFRCT. Revascularization and major MACEs were more frequent in patients with FFRCT ≤0.80 compared to those with FFRCT >0.80 (RR 6.87; 95% CI: 5.59–8.45; *p* < 0.001 and RR: 1.81; 95% CI: 0.96–3.43; *p* = 0.06, respectively) (186, 187).

## Conclusions

The field of coronary angiography has been continuously evolving since its introduction in clinical practice. Efforts have been made to develop new non-invasive modalities for precise assessment of coronary artery disease, moving from visual quantification of lesion severity to functional assessment. The development of QFR and FFR_CT_ are two pioneering examples. Further research is needed to improve these technologies, especially in the use of artificial intelligence. The development of AI promises to shorten the time required and decrease costs and resources in routine coronary angiography in the years to come. Future studies and trials will be needed to assess the major clinical outcomes and cost-effectiveness in the application of these upcoming technologies.

## Supplementary material

Supplementary Figure S1 Angiographic late lumen loss as a predictor of clinical events at follow-up. Upper panel: Logistic regression curve to predict probability of target lesion revascularization and actual incidence of target lesion revascularization. (**A**) The probability curves generated by logistic regression analysis to estimate the probability of target lesion revascularization at 2 years are presented with the actual incidence of target lesion revascularization in the current cohort (n = 2,426). Histogram shows the distribution of in-stent LLL in the current cohort. (**B**) Magnified diagram of (**A**) to focus on LLL between 0.1 and 0.6 mm. DES, drug-eluting stent; LLL, late lumen loss; TLR, target lesion revascularization [Reproduced from Asano et al. (38)]. Lower panel: Kaplan–Meier (**A**) estimates of target lesion revascularization after follow-up angiography [Reproduced from Asano et al. (38)].

Supplementary Figure S2 Diagnostic performance of angiography-derived fractional flow reserve: a systematic review and Bayesian meta-analysis. Individual studies and summary point estimates for sensitivity (**A**) and specificity (**B**). Estimates within 95% credible intervals are shown. At the bottom, a summary estimate combining all studies is provided. FN, false negatives; FP, false positives; TN, true negatives; TP, true positives [Reproduced from Collet et al. (104)].

Supplementary Figure S3 Prediction of 2-year VOCEs for the whole population of the ongoing Multivessel TALENT trial based on post-PCI QFR results of half of the population. Two different offline angiography-derived FFR software were used depending on whether there were one or two angiographic views available to obtain the pre- and post-PCI QFR in the maximum number of participants in the ongoing Multivessel TALENT trial before event adjudication. In cases where only baseline or post-procedural QFR were available, µFR analysis was added, pre- or post-PCI, in the same vessel. Eleven vessels were analyzed with both techniques post-procedure. An optimal post-PCI QFR was obtained in 76% of the treated vessels. Based on the relationship between post-PCI QFR and 2-year VOCEs in the SYNTAX II trial, the predicted 2-year VOCE prevalence in the entire Multivessel TALENT trial population was 6.1%, with a boundary of 4.8% to 7.4%. FFR, fractional flow reserve; PCI, percutaneous coronary intervention; QFR, quantitative flow ratio; VOCE, vessel-oriented composite endpoint; µFR, Murray law-based QFR [Reproduced from Kageyama et al. (123)].
