# Supplementary figures and images for: Coronary angiography: a review of the state of the art and the evolution of angiography in cardio therapeutics
Source: Front Cardiovasc Med. 2024 Nov 25;11:1468888. doi: 10.3389/fcvm.2024.1468888 (PMC11625592; doi:10.3389/fcvm.2024.1468888)

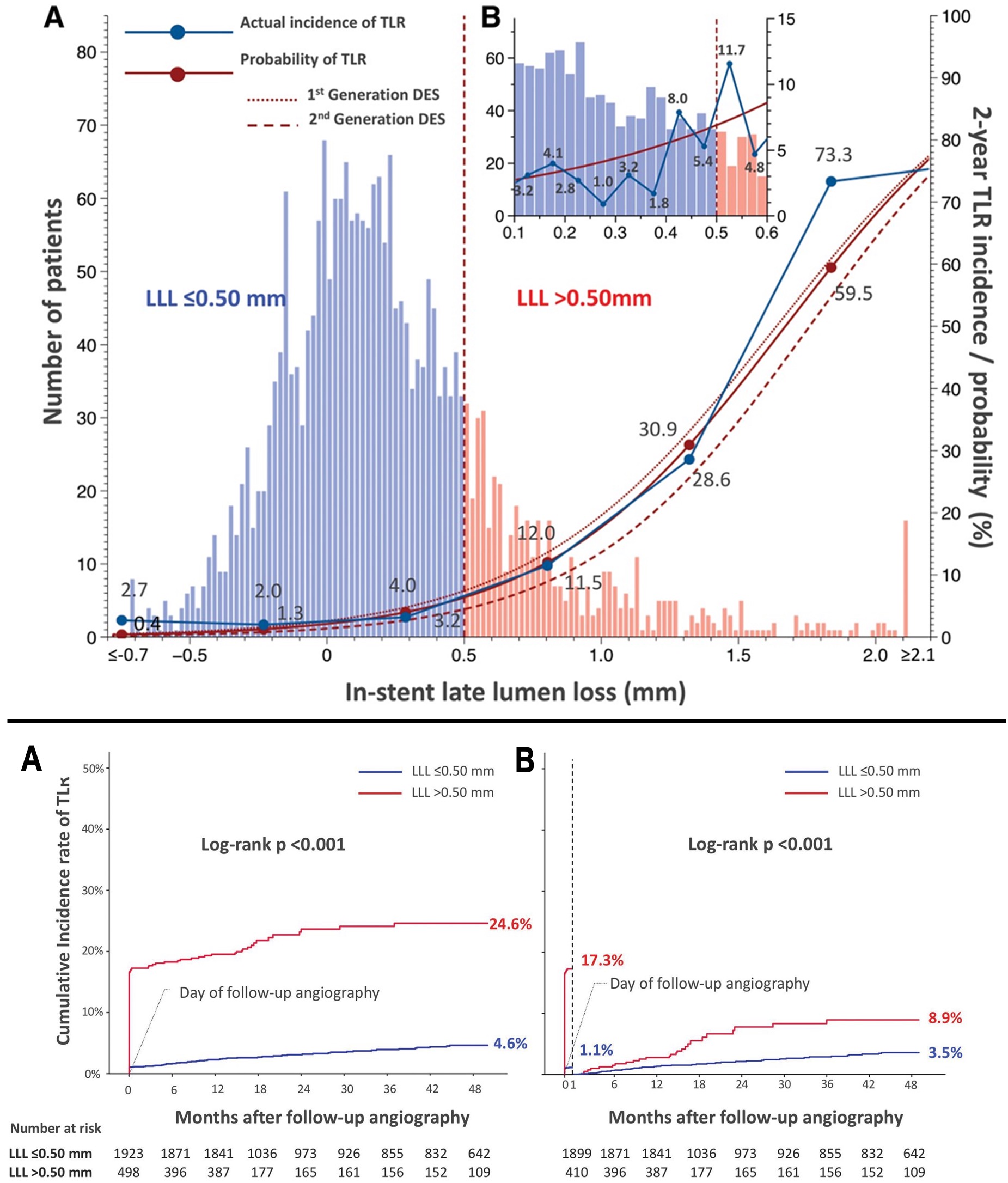

Supplement: Supplementary Figure S1 [file Image1.jpeg]

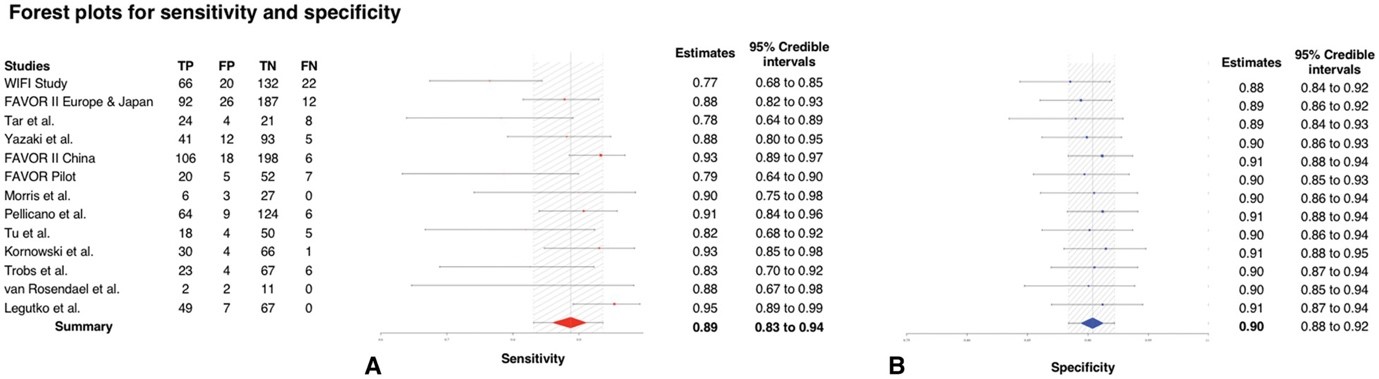

Supplement: Supplementary Figure S2 [file Image2.jpeg]

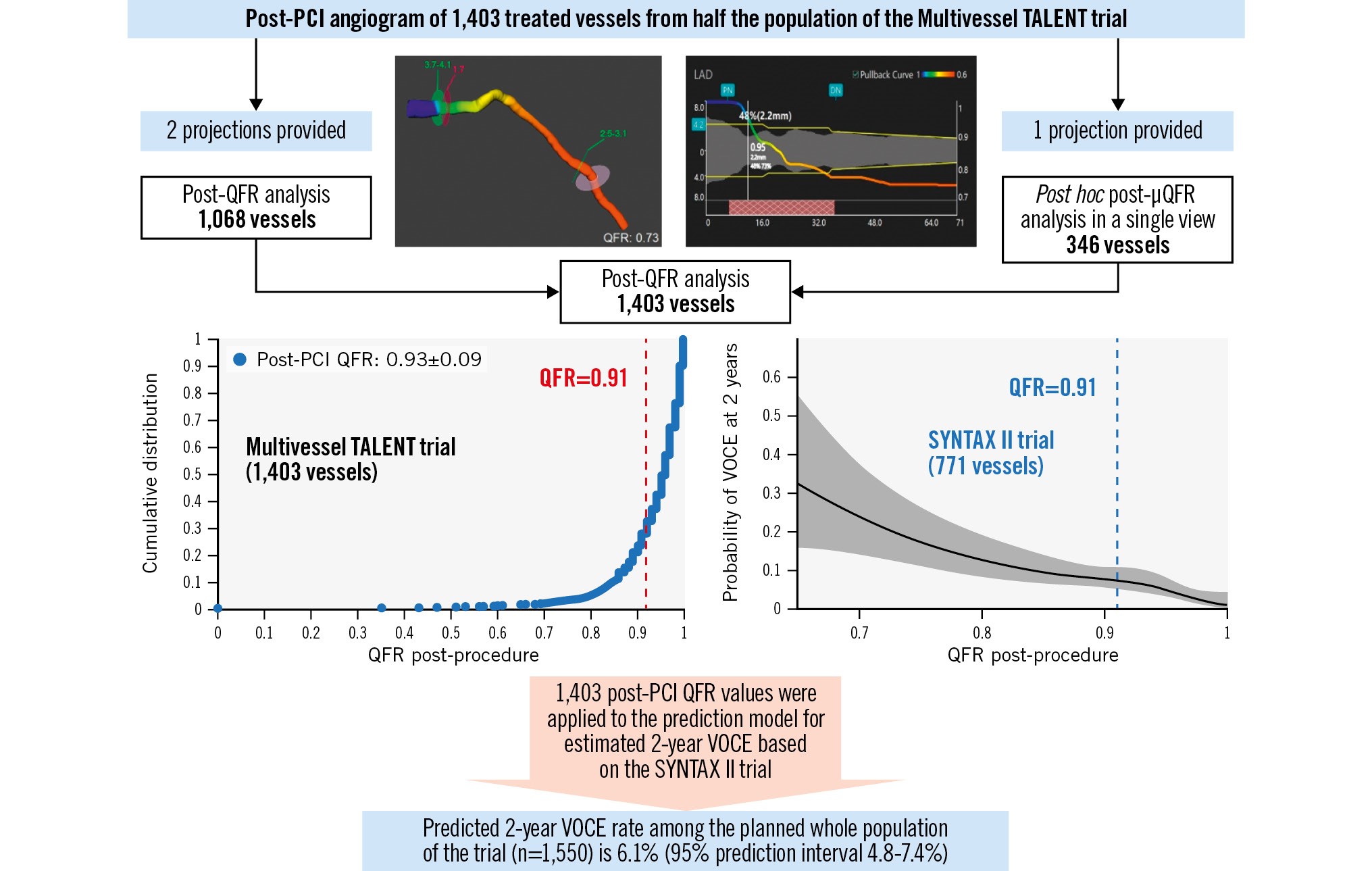

Supplement: Supplementary Figure S3 [file Image3.jpeg]
