# Supplementary material for: Coronary angiography: a review of the state of the art and the evolution of angiography in cardio therapeutics
Source: Front Cardiovasc Med. 2024 Nov 25;11:1468888. doi: 10.3389/fcvm.2024.1468888 (PMC11625592; doi:10.3389/fcvm.2024.1468888)
Supplement: Supplementary file 4 [file Datasheet1.pdf]

Coronary angiography: State of the art review

Glimpse into the past, current and future - the evolution of angiography in Cardio Therapeutics

Angiography based FFR systems

1) Quantitative flow ratio:

The Quantitative flow ratio (QFR®) is calculated using the QAngio XA 3D 2.1 software package (Medis Medical Imaging System BV, Leiden, The Netherlands) by estimating the pressure drop using simplified equations [1-3] based on models from Young [4] and Kirkeeide [5]. QFR analysis requires two diagnostic angiographic projections ≥ 25° apart. The foreshortening of the target vessel on each projection is reported as a percentage. The target vessel is reconstructed based on automatic contour detection without assessment of fractal division. Minimal contour adjustment areallowed and documented. The estimated contrast flow velocity (CFV) is performed automatically frame counting analysis. Hyperemic flow velocity (HFV) is modeled from contrast flow as $HFV=a_0+a_1\times CFV+a_2\times CFV^2$, where a_0=0.10, a_1=1.55, and a_2= -0.93.[2] QFR is the first commercially available software to be CE-marked and FDA-approved. Prior studies have shown a good correlation between QFR and FFR values in angiographically intermediate lesions, with a good diagnostic accuracy of QFR for assessing functional stenosis severity.[6] Two prospective, multicentre studies (Angiographic quantitative flow ratio-guided coronary intervention - FAVOR II China and FAVOR II Europe-Japan) have reported good diagnostic accuracies of QFR both at patient and vessel levels[7] and better sensitivity and specificity than 2D-QCA in assessing functional stenosis relevance[8]. In a patient-level meta-analysis of 16 high-quality studies comparing FFR and QFR, QFR demonstrated good positive and excellent negative predictive values in ascertaining the functional relevance of coronary stenoses with a cut-off FFR of ≤0.80.[9] The results of the FAVOR III Europe outcome trial in 2000 patients will be presented at the ESC 2024.

2) Vessel fractional flow reserve (vFFR)

vFFR computation uses the CAAS workstation (Pie Medical Imaging, Maastricht, The Netherlands) and is based on the mathematical approach from Lance Gould [5] and Kirkeeide.[4] vFFR requires two projections with ≥ 30° difference angulation to perform coronary 3D reconstruction.[2,10,11] Aortic root pressure is used as an input boundary condition. vFFR calculates pressure drop by applying physical laws, that includes viscous

resistance and separation loss effects, to 3D reconstructed geometry. The pressure drop calculation by vFFR includes patient specific aortic pressure as measured during the catheterisation procedure. Maximum hyperaemic blood flow was empirically determined from clinical data and proximal coronary velocity is assumed to be preserved along the coronary of interest which is adapted based on the patient-specific aortic rest pressure and the 3D reconstructed geometry. vFFR is CE-marked in Europe, FDA-approved, and commercially available for clinical use. FAST III study, is a multicenter, randomized trial evaluating MACE at 1 year in vFFR-guided PCI vs FFR-guided PCI.[12] The LIPSIA STRATEGY study (NCT03497637) is an ongoing German multi-center prospective trial at 7 sites enrolling 2000 patients investigating a vFFR vs FFR guided stenting strategy. The FAST OCT study (NCT04683133) is a prospective, multicenter, single-arm study to define the association between vFFR and OCT findings in intermediate coronary artery lesions in patients presenting with NSTE-ACS. FAST STEMI I study, showed that In STEMI patients with multivessel disease, discordance between vFFR reclassification and treatment strategy was observed in 21.1% of non-culprit vessels with an intermediate lesion and was associated with increased vessel-related adverse events.[13] The vFFR HeartTeam study, showed that three vessel vFFR screening indicated discordance between vFFR confirmed lesion significance and revascularisation in 29.8% of the patients.[14] FAST Post Study, study showed that 3D-QCA derived post PCI vFFR correlates well with invasively measured microcatheter based FFR and has a high diagnostic accuracy to detect FFR <0.90 with low inter-observer variability.[2] The FAST II (Fast Assessment of STenosis severity) study was a prospective observational multicentre study designed to evaluate the diagnostic accuracy of vFFR compared to the reference standard (pressure wire-based FFR ≤0.80). A total of 334 patients from six centres were enrolled. Both site-determined and blinded independent core lab vFFR measurements were compared to FFR. 3D-QCA-based vFFR has excellent diagnostic performance to detect FFR ≤0.80.[15]

3) Angio-iFR/FFR Computation

The Angio-iFR algorithm uses a lumped parameter fluid dynamics model employing an electric-hydraulic analogy [16,17]; the coronary hydraulic network model is created as an electrical circuit “powered” by the heart. Details of the computation of Angio-iFR/FFR measurement are included in the Supplementary File. Briefly, the basic components of the coronary vasculature are modeled as follows: volumetric blood flow (Q),

pressure (P), and the vascular resistance including coronary lesion (R) equates to electrical current (I), voltage (V), and resistance (R), respectively. In the hydraulic analog, a dynamic pump pushes a viscous fluid through pipes with various degrees of blockage or constriction.

The pressure drop associated with fluid passing through each segment can be derived using Poiseuille's Law, Darcy-Weisbach friction, and Borda-Carnot expansion loss variables. Branching arteries are modeled as outlets reducing the local volumetric flow rate in the primary vessel; the microvasculature is modeled as an outlet resistor; and the venous system is treated as the electrical ground – or the termination of the circuit. This lumped parameter modeling approach is computationally efficient, and enables real-time analysis during the coronary catheterization.

The software is composed of two separate models for estimating the functional significance of a lesion by FFR and iFR separately. For simulation of FFR and iFR, different boundary conditions are used. For the estimation of FFR, the hyperemic flow state is assumed, myocardial resistance is taken to be minimal and independent of the lesions, and all flow variation is thus considered to be associated with the epicardial lesion. For the estimation of iFR, the myocardial resistance varies with the lesion resistance and the simulated flow is almost independent of the resistance associated with the lesion until the lesion becomes critically narrowed.

4) Coronary angiography-derived fractional flow reserve (caFFR)

caFFR analysis, requires the Flash Angio software (Rainmed Ltd, Suzhou, China),and uses a computational pressure-flow dynamics method to solve the Navier-Stokes equation and compute the pressure drop along the coronary artery of interest. [18,19] caFFR requires two projections ≥ 30 degrees apart. An anisotropic filter and hessian matrix are applied for image processing. Foreshortening is accounted in the analysis but not reported. Contour detection is based on the active contour method with the 2D vessel diameter calculated using centerline normal vector interaction with segmentation contour. A circular model is used for vessel reconstruction using coordinate transformation and the standard deviation of corresponding 2D diameters from two projections for the 3D diameter. Flow velocity is determined using frame counting analysis, and HFV is calculated by adding a constant obtained from a preliminary retrospective study.[19] The aortic pressure and HFV are used as boundary conditions.

5) Murray law-based quantitative flow ratio (2DμFR and 3D μFR)

μFR is computed using empirical fluid dynamic equations from Kirkeeide, [20] a single projection, the Pulse software (Pulse Medical Imaging Technology, Shanghai Co., Ltd., Shanghai, China), and the following methodology: (a) the target vessel is delineated during contrast injection; (b) the contrast flow velocity is derived using AI-facilitated frame count analysis and converted to HFV based on fluid dynamics equations;[20] (c) the frame with the sharpest lumen contour of the stenotic segment is used to delineate the lumen boundary of the target vessel and its major side branches (lumen diameter ≥1.0 mm); step-down RVD is computed based on Murray bifurcation fractal law [21];(d) finally, the pressure drop is calculated using the above-mentioned hyperemic flow as the boundary condition. In μFR analysis, the projection with the least vessel foreshortening and least side branch overlap is selected from the two available projections, which are used in the other version of the software dedicated to a two-projection analysis. This latter method provides a two-projection-based 3D μFR, which was considered the fifth method of computing angio-FFR in this study. A recent report demonstrated that computation of μQFR from a single angiographic view had comparable diagnostic performance as 2-view 3D-μQFR with an AUC of 0.957.[22] It has shown a high -diagnostic performance in predicting wire-based FFR values with an analyzability of 100% in the validation of the cohort of the FAVOR II China study. There are a few methodological queries raised though. Firstly, it is unclear how to choose the optimal view, especially in the case of eccentric lesions as the diagnostic accuracy is known to decrease if the projection is suboptimal. Secondly, it is challenging to acquire a good angiographic view with visualization of all side branches without overlapping or shortening. On the other hand, this novel approach allows for automation frame counting as well as lumen contour delineation.

6) FFR Angio (Cath Works)

The primary element of the angiogram-based FFR measurement is the 3-dimensional (3D) rebuilding of the coronary tree. The reconstruction is based on the known geometry of ≥3 projections from single-plane angiograms and uses epipolar ray tracing together with mathematical constraints enforcing the tree's structure. After 3D reconstruction the system scans the entire reconstructed tree in 3D and analyses each branch as well as each bifurcation (or trifurcation), looking for narrowed regions. A hemodynamic evaluation follows, where the contribution of each narrowing to the total resistance to flow is taken into account and a

subsequent lumped model is built. This allows pressure drops and flow rates to be estimated. The accumulated volume of the coronary tree and the total coronary length, calculated from a reconstruction of its geometry, enable an estimation of normal supply through an assessment of the microcirculatory bed resistance. The solution of the lumped model based on the inlet and outlet boundary conditions allows us to evaluate ratios of flow rate for stenosed versus "healthy" coronary trees. This first-in-human study indicated high reproducibility and diagnostic accuracy of FFR_{angio} compared with invasive FFR.[23] In a pooled analysis of 5 prospective cohort studies involving 700 lesions from 588 patients, when using a binary cutoff FFR value of 0.80, FFRangio showed a sensitivity of 91%, a specificity of 94%, and a diagnostic accuracy of 93%. The mean difference between FFR and FFRangio was 0.00 ± 0.12.[24] The correlation coefficient between FFR and FFRangio was 0.83 ($p < 0.001$). The C-statistic for FFRangio was 0.95 ($p < 0.001$). The accuracy of FFRangio was consistent across all subgroups examined.[24]

7) Auto Cath FFR:

It is a novel non-invasive software that can instantaneously produce FFR values derived completely from artificial intelligence (AI) -based algorithms. AutocathFFR, utilizesConvolutional Neural Networks, for video analysis . The software progresses through a multi-component algorithm allowing for vessel segmentation and lesions detection followed by AI-FFR calculations within less than a minute. In a retrospective, three-center study comparing AI-FFR values with invasive pressure wire–derived FFR which analysed a total of 304 vessels from 297 patients, mean invasive FFR was 0.86 vs. 0.85 AI-FFR (mean difference: −0.005, $P = 0.159$).[25] The diagnostic performance of AI-FFR demonstrated a sensitivity of 91%, specificity of 95%, positive predictive value of 83% and negative predictive value of 97%. The overall accuracy was 94% and the area under curve was 0.93 (95% CI 0.88–0.97).[25] 105 lesions had a borderline positive/negative FFR (FFR = 0.75–0.85); in this sub-group, AI-FFR demonstrated sensitivity of 95%, and specificity of 94%, with an AUC of 0.94 (95% CI 88.2–98.0). AI-FFR calculation time was 37.5 ± 7.4 s for eachstudied case .[25]

8) AngioFFR

The non-commercial prototype software Angiographic FFR (angioFFR; Siemens Healthcare

GmbH, Forchheim, Germany), is one of the first artificial intelligence (AI)-based angiography-derived FFR applications. AngioFFR uses an upfront-trained machine learning model with >27,000 synthetic geometries with angioFFR values that are based on 3-dimensional (3D) computational fluid dynamics. In a clinical setting, angioFFR values can then be extracted from the upfront-trained data without complex 3D CFD analysis.[26] In a single-center study conducted between November 2018 and February 2020 involving 200 patients and 253 vessels with 30-90% angiographic stenoses the accuracy of angioFFR was 87.7% (95% confidence interval [CI] 83.1-91.5%), with a sensitivity of 76.8% (95% CI 67.1-84.9%), specificity of 94.3% (95% CI 89.5-97.4%), and area under the curve of 0.90 (95% CI 0.86-0.93%).[26]

References.

1. Tu S, Westra J, Yang J et al. Diagnostic accuracy of fast computational approaches to derive fractional flow reserve from diagnostic coronary angiography: the international multicenter FAVOR pilot study. Cardiovascular Interventions 2016;9:2024-2035.
2. Masdjedi K, van Zandvoort LJ, Balbi MM et al. Validation of a three-dimensional quantitative coronary angiography-based software to calculate fractional flow reserve: the FAST study. EuroIntervention 2020;16:591-599.
3. Young DF, Tsai FY. Flow characteristics in models of arterial stenoses—I. Steady flow. Journal of biomechanics 1973;6:395-410.
4. Gould K, Kelley K, Bolson E. Experimental validation of quantitative coronary arteriography for determining pressure-flow characteristics of coronary stenosis. Circulation 1982;66:930-937.
5. Kirkeeide RL, Gould KL, Parsel L. Assessment of coronary stenoses by myocardial perfusion imaging during pharmacologic coronary vasodilation. VII. Validation of coronary flow reserve as a single integrated functional measure of stenosis severity reflecting all its geometric dimensions. Journal of the American College of Cardiology 1986;7:103-113.
6. Faria D, Hennessey B, Shabbir A et al. Functional coronary angiography for the assessment of the epicardial vessels and the microcirculation. EuroIntervention 2023;19:203-221.

7. Xu B, Tu S, Qiao S et al. Diagnostic Accuracy of Angiography-Based Quantitative Flow Ratio Measurements for Online Assessment of Coronary Stenosis. J Am Coll Cardiol 2017;70:3077-3087.
8. Westra J, Andersen BK, Campo G et al. Diagnostic performance of in-procedure angiography-derived quantitative flow reserve compared to pressure-derived fractional flow reserve: the FAVOR II Europe-Japan study. Journal of the American Heart Association 2018;7:e009603.
9. Westra J, Tu S, Campo G et al. Diagnostic performance of quantitative flow ratio in prospectively enrolled patients: An individual patient-data meta-analysis. Catheter Cardiovasc Interv 2019;94:693-701.
10. Tu S, Ding D, Chang Y, Li C, Wijns W, Xu B. Diagnostic accuracy of quantitative flow ratio for assessment of coronary stenosis significance from a single angiographic view: a novel method based on bifurcation fractal law. Catheterization and Cardiovascular Interventions 2021;97:1040-1047.
11. Huo Y, Svendsen M, Choy JS, Zhang Z-D, Kassab GS. A validated predictive model of coronary fractional flow reserve. Journal of The Royal Society Interface 2012;9:1325-1338.
12. Scoccia A, Byrne RA, Banning AP et al. Fractional flow reserve or 3D-quantitative-coronary-angiography based vessel-FFR guided revascularization. Rationale and study design of the prospective randomized fast III trial. Am Heart J 2023;260:1-8.
13. Groenland FTW, Huang J, Scoccia A et al. Vessel fractional flow reserve-based non-culprit lesion reclassification in patients with ST-segment elevation myocardial infarction: Impact on treatment strategy and clinical outcome (FAST STEMI I study). Int J Cardiol 2023;373:33-38.
14. Tomaniak M, Masdjedi K, Neleman T et al. Three-dimensional QCA-based vessel fractional flow reserve (vFFR) in Heart Team decision-making: a multicentre, retrospective, cohort study. BMJ Open 2022;12:e054202.

15. Masdjedi K, Tanaka N, Van Belle E et al. Vessel fractional flow reserve (vFFR) for the assessment of stenosis severity: the FAST II study. EuroIntervention 2022;17:1498-1505.
16. Donnelly PM, Kolossvary M, Karady J et al. Experience With an On-Site Coronary Computed Tomography-Derived Fractional Flow Reserve Algorithm for the Assessment of Intermediate Coronary Stenoses. Am J Cardiol 2018;121:9-13.
17. van Hamersvelt RW, Voskuil M, de Jong PA, Willemink MJ, Isgum I, Leiner T. Diagnostic Performance of On-Site Coronary CT Angiography-derived Fractional Flow Reserve Based on Patient-specific Lumped Parameter Models. Radiol Cardiothorac Imaging 2019;1:e190036.
18. Kornowski R, Lavi I, Pellicano M et al. Fractional flow reserve derived from routine coronary angiograms. Journal of the American College of Cardiology 2016;68:2235-2237.
19. Murray CD. The physiological principle of minimum work: I. The vascular system and the cost of blood volume. Proceedings of the National Academy of Sciences 1926;12:207-214.
20. Schuurbiers JC, Lopez NG, Ligthart J et al. In vivo validation of CAAS QCA-3D coronary reconstruction using fusion of angiography and intravascular ultrasound (ANGUS). Catheterization and Cardiovascular interventions 2009;73:620-626.
21. Nijjer SS, de Waard GA, Sen S et al. Coronary pressure and flow relationships in humans: phasic analysis of normal and pathological vessels and the implications for stenosis assessment: a report from the Iberian–Dutch–English (IDEAL) collaborators. European heart journal 2015;37:2069-2080.
22. Ding D, Tu S, Chang Y, Li C, Xu B, Wijns W. Quantitative flow ratio based on Murray fractal law: accuracy of single versus two angiographic views. Journal of the Society for Cardiovascular Angiography & Interventions 2022;1:100399.
23. Kornowski R, Lavi I, Pellicano M et al. Fractional Flow Reserve Derived From Routine Coronary Angiograms. J Am Coll Cardiol 2016;68:2235-2237.

24. Witberg G, De Bruyne B, Fearon WF et al. Diagnostic Performance of Angiogram-Derived Fractional Flow Reserve: A Pooled Analysis of 5 Prospective Cohort Studies. JACC Cardiovasc Interv 2020;13:488-497.
25. Ben-Assa E, Abu Salman A, Cafri C et al. Performance of a novel artificial intelligence software developed to derive coronary fractional flow reserve values from diagnostic angiograms. Coron Artery Dis 2023;34:533-541.
26. Omori H, Kawase Y, Mizukami T et al. Diagnostic Accuracy of Artificial Intelligence-Based Angiography-Derived Fractional Flow Reserve Using Pressure Wire-Based Fractional Flow Reserve as a Reference. Circ J 2023;87:783-790.
